# Supplementary material for: Association between tuberculosis and pregnancy outcomes: a retrospective cohort study of women in Cape Town, South Africa
Source: BMJ Open. 2024 Feb 7;14(2):e081209. doi: 10.1136/bmjopen-2023-081209 (PMC10860026; doi:10.1136/bmjopen-2023-081209)
Supplement: Supplementary data [file bmjopen-2023-081209supp001.pdf]

The association between tuberculosis and pregnancy outcomes: A retrospective cohort study of women in Cape Town, South Africa.

Authors

Sue-Ann Meehan[1*], Anneke C. Hesseling[1], Arne von Delft[2,3], Florian M. Marx[1,4], Jennifer A. Hughes[1], Peter Bock[1], Aduragbemi Banke-Thomas[5,6], Rory Dunbar[1], Florence Phelanyane[2,3], Mariette Smith[2,3], Muhammad Osman[1,5]

*Corresponding author

Email address: sueannm@sun.ac.za (SM)

Affiliations:

[1]Desmond Tutu TB Centre, Department of Paediatrics and Child Health, Faculty of Medicine and Health Sciences, Stellenbosch University

[2]Centre for Infectious Disease Epidemiology and Research (CIDER), School of Public Health and Family Medicine, Faculty of Health Sciences, University of Cape Town

[3]Health Intelligence Directorate, Western Cape Government, Department of Health and Wellness

[4]Division of Infectious Disease and Tropical Medicine, Centre for Infectious Diseases, Heidelberg University Hospital, Heidelberg, Germany

[5]School of Human Sciences, Faculty of Education, Health and Human Sciences, University of Greenwich, London, United Kingdom

[6]Faculty of Epidemiology and Population Health, London School of Hygiene and Tropical Medicine, London, United Kingdom

Supplementary Table S1: Demographic and clinical characteristics among pregnant women with tuberculosis and HIV, stratified by pregnancy status at the time antiretroviral treatment was initiated (n=130).

	Total N (%)	Pregnant at ART start n (%)	
	**(N=130)**	**Yes (n=56), 43%**	**No (n=74), 57%**
**CD4 count*, median (IQR)**	235 (127-423)	231 (100-352)	260 (134-468)
**Age**			
14-17 years	3 (2.3)	0 (0)	3 (4.1)
18-24 years	25 (19.2)	9 (16.1)	16 (21.6)
25-34 years	82 (63.1)	39 (69.6)	43 (58.1)
35-44 years	20 (15.4)	8 (14.3)	12 (16.2)
**Pregnancy recorded in relation to TB diagnosis**			
Pregnant before TB diagnosed	33 (25.4)	14 (25.0)	19 (25.7)
Pregnant after TB diagnosed	97 (74.6)	42 (75.0)	55 (74.3)
**TB treatment at ART start**			
yes	37 (28.5)	21 (37.5)	16 (21.6)
no	93 (71.5)	35 (62.5)	58 (78.4)
**Viral suppression ever**			
yes	115 (88.5)	48 (85.7)	67 (90.5)
no	15 (11.5)	8 (14.3)	7 (9.5)
**Last VL suppressed**			
yes	97 (74.6)	42 (75.0)	55 (74.3)
no	33 (25.4)	14 (25.0)	19 (25.7)
**Time known to ANC services (days) (%)**			
0-30	33 (25.4)	17 (30.4)	16 (21.6)
31-120	41 (31.5)	19 (33.9)	22 (29.7)
≥ 121	56 (43.1)	20 (35.7)	36 (48.6)
**Pregnancy Outcome (%)**			
Good	66 (50.8)	30 (53.6)	36 (48.6)
Adverse	40 (30.8)	17 (30.4)	23 (31.1)
unknown	24 (18.5)	9 (16.1)	15 (20.3)

*CD4 count closest to date of TB diagnosis

ART: antiretroviral treatment, VL: viral load, ANC: antenatal care

Meehan S-A, *et al. BMJ Open* 2024; 14:e081209. doi: 10.1136/bmjopen-2023-081209
