# Supplementary material for: Association between tuberculosis and pregnancy outcomes: a retrospective cohort study of women in Cape Town, South Africa
Source: BMJ Open. 2024 Feb 7;14(2):e081209. doi: 10.1136/bmjopen-2023-081209 (PMC10860026; doi:10.1136/bmjopen-2023-081209)
Supplement: Author's manuscript [file bmjopen-2023-081209.draft_revisions.pdf]

BMJ Open is committed to open peer review. As part of this commitment we make the peer review history of every article we publish publicly available.

When an article is published we post the peer reviewers' comments and the authors' responses online. We also post the versions of the paper that were used during peer review. These are the versions that the peer review comments apply to.

The versions of the paper that follow are the versions that were submitted during the peer review process. They are not the versions of record or the final published versions. They should not be cited or distributed as the published version of this manuscript.

BMJ Open is an open access journal and the full, final, typeset and author-corrected version of record of the manuscript is available on our site with no access controls, subscription charges or pay-per-view fees (http://bmjopen.bmj.com).

If you have any questions on BMJ Open's open peer review process please email info.bmjopen@bmj.com

BMJ Open

Pregnant women with tuberculosis have high rates of unfavourable pregnancy outcomes irrespective of HIV status in Cape Town, South Africa

Journal:	*BMJ Open*
Manuscript ID	bmjopen-2023-081209
Article Type:	Original research
Date Submitted by the Author:	21-Oct-2023
Complete List of Authors:	Meehan, S; Stellenbosch University Faculty of Medicine and Health Sciences, Desmond Tutu TB Centre, Department of Paediatrics and Child Health<br>Hesseling, A C; Stellenbosch University Faculty of Medicine and Health Sciences, Desmond Tutu TB centre, Department of Paediatrics and Child Health<br>von Delft, Arne; University of Cape Town Department of Medicine, Centre for Infectious Disease Epidemiology and Research (CIDER), School of Public Health and Family Medicine; Western Cape Department of Health, Health Intelligence Directorate<br>Marx, Florian M.; Stellenbosch University Faculty of Medicine and Health Sciences, Desmond Tutu TB centre, Department of Paediatrics and Child Health; Heidelberg university hospital, Division of Infectious Disease and Tropical Medicine, Centre for Infectious Diseases<br>Hughes, Jennifer A.; Stellenbosch University, Desmond Tutu TB Centre, Department of Paediatrics and Child Health<br>Bock, Peter; Stellenbosch University Faculty of Medicine and Health Sciences, Desmond Tutu TB Centre, Department of Paediatrics and Child Health<br>Banke-Thomas, Aduragbemi; University of Greenwich; London School of Hygiene & Tropical Medicine, Faculty of Epidemiology and Population Health<br>Dunbar, R.; University of Stellenbosch, Desmond Tutu TB Centre, Department of Paediatrics and Child Health<br>Phelanyane, Florence; University of Cape Town Faculty of Health Sciences, Centre for Infectious Disease Epidemiology and Research (CIDER), School of Public Health and Family Medicine; Western Cape Department of Health, Health Intelligence Directorate, Department of Health and Wellness<br>Smith, Mariette; Western Cape Department of Health, Health Intelligence Directorate, Department of Health and Wellness ; University of Cape Town Faculty of Health Sciences, Centre for Infectious Disease Epidemiology and Research (CIDER), School of Public Health and Family Medicine<br>Osman, Muhammad; Stellenbosch University Faculty of Medicine and Health Sciences, Desmond Tutu TB Centre, Department of Paediatrics and Child Health; University of Greenwich, School of Human Sciences, Faculty of Education, Health and Human Sciences

Keywords:	Tuberculosis < INFECTIOUS DISEASES, HIV & AIDS < INFECTIOUS DISEASES, Public health < INFECTIOUS DISEASES, Epidemiology < INFECTIOUS DISEASES

SCHOLARONE™
Manuscripts

I, the Submitting Author has the right to grant and does grant on behalf of all authors of the Work (as defined in the below author licence), an exclusive licence and/or a non-exclusive licence for contributions from authors who are: i) UK Crown employees; ii) where BMJ has agreed a CC-BY licence shall apply, and/or iii) in accordance with the terms applicable for US Federal Government officers or employees acting as part of their official duties; on a worldwide, perpetual, irrevocable, royalty-free basis to BMJ Publishing Group Ltd ("BMJ") its licensees and where the relevant Journal is co-owned by BMJ to the co-owners of the Journal, to publish the Work in this journal and any other BMJ products and to exploit all rights, as set out in our licence.

The Submitting Author accepts and understands that any supply made under these terms is made by BMJ to the Submitting Author unless you are acting as an employee on behalf of your employer or a postgraduate student of an affiliated institution which is paying any applicable article publishing charge ("APC") for Open Access articles. Where the Submitting Author wishes to make the Work available on an Open Access basis (and intends to pay the relevant APC), the terms of reuse of such Open Access shall be governed by a Creative Commons licence – details of these licences and which Creative Commons licence will apply to this Work are set out in our licence referred to above.

Other than as permitted in any relevant BMJ Author's Self Archiving Policies, I confirm this Work has not been accepted for publication elsewhere, is not being considered for publication elsewhere and does not duplicate material already published. I confirm all authors consent to publication of this Work and authorise the granting of this licence.

For peer review only

Pregnant women with tuberculosis have high rates of unfavourable pregnancy outcomes irrespective of HIV status in Cape Town, South Africa

Authors

Sue-Ann Meehan[1*], Anneke C. Hesseling[1], Arne von Delft[2,3], Florian M. Marx[1,4], Jennifer A. Hughes[1], Peter Bock[1], Aduragbemi Banke-Thomas[5,6], Rory Dunbar[1], Florence Phelanyane[2,3], Mariette Smith[2,3], Muhammad Osman[1,5]

*Corresponding author

Email address: sueannm@sun.ac.za (SM)

Affiliations:

[1]Desmond Tutu TB Centre, Department of Paediatrics and Child Health, Faculty of Medicine and Health Sciences, Stellenbosch University

[2]Centre for Infectious Disease Epidemiology and Research (CIDER), School of Public Health and Family Medicine, Faculty of Health Sciences, University of Cape Town

[3]Health Intelligence Directorate, Western Cape Government, Department of Health and Wellness

[4]Division of Infectious Disease and Tropical Medicine, Centre for Infectious Diseases, Heidelberg University Hospital, Heidelberg, Germany

[5]School of Human Sciences, Faculty of Education, Health and Human Sciences, University of Greenwich, London, United Kingdom

[6]Faculty of Epidemiology and Population Health, London School of Hygiene and Tropical Medicine, London, United Kingdom

Running Head: Pregnancy outcomes among women with TB

Key words: Maternal TB, pregnancy outcomes, TB outcomes, linkage to care, HIV

Journal: BMJ Global health

Word count: Abstract: 284/300 Main text: 4141/5000

For peer review only - http://bmjopen.bmj.com/site/about/guidelines.xhtml

Abstract

Background

Tuberculosis remains a leading cause of mortality among women of childbearing age and a significant contributor to maternal mortality. Pregnant women with tuberculosis are at high risk of adverse pregnancy outcomes. This study aimed to determine risk factors for an unfavourable pregnancy outcome among pregnant women diagnosed with tuberculosis,

Methods

Using tuberculosis programmatic data, this retrospective analysis included all women who were routinely diagnosed with tuberculosis in the public sector between October 2018 and March 2020 in two health sub-districts of Cape Town, and who were documented to be pregnant during their tuberculosis episode. Unfavourable pregnancy outcome was defined as either a live birth of an infant weighing <2500g and/or with a gestation period <37 weeks or as stillbirth, miscarriage, termination of pregnancy, maternal or early neonatal death. Logistic regression was used to determine risk factors for unfavourable pregnancy outcome.

Results

Of 248 pregnant women, half (52%) were living with HIV; all were on antiretroviral therapy (ART) at the time of their tuberculosis diagnosis. Pregnancy outcomes were documented in 215 (87%) women, of whom 74 (34%) had an unfavourable pregnancy outcome. Being older (35-44 years vs 25-34 years (aOR: 3.99; 95% CI:1.37-11.57), living with HIV (aOR: 2.72; 95%CI: 0.99-4.63), having an unfavourable TB outcome (aOR 2.29; 95% CI: 1.03-5.08), and having presented to antenatal services ≤1 month prior to delivery (aOR 10.57: 95% CI:4.01-27.89) were associated with higher odds of unfavourable pregnancy outcome.

Conclusions

Pregnant women with TB are a highly vulnerable group who need additional support prior to, during and after TB treatment to improve TB treatment otucomes and pregnancy outcomes, regardless of HIV status. Pregnancy status is an important indicator which should be considered for inclusion in TB registries going forward.

For peer review only - http://bmjopen.bmj.com/site/about/guidelines.xhtml

Key messages

What is already known on this topic:

- Women with TB and TB/HIV are at high risk of poor pregnancy outcomes.
- Pregnancy status is not routinely captured or reported as a TB programme indicator
- There are limited data on TB treatment outcomes amongst pregnant women

What this study adds:

- Using an expanded definition of an unfavourable TB treatment outcome, to include loss to follow up prior to linkage to TB care, a third of pregnant women had an unfavourable TB outcome
- Overall 34% of women with a known pregnancy outcome had an unfavourable pregnancy outcome, higher among women who had unfavourable TB outcomes (40%) vs. favourable TB outcome (24%), irrespective of HIV status
- Having an unfavourable TB outcome was associated with higher odds of unfavourable pregnancy outcome.

How this study might affect research, practice or policy:

- Pregnant women with TB are a highly vulnerable group who need additional support prior to, during and after TB treatment to reduce poor pregnancy outcomes, even those who are HIV-negative.
- Recording of pregnancy status should be a key indicator piloted as part of TB treatment registries, as this would result in better quality data and offer opportunites for earlier intervention and support of pregnant and postpartum women with TB.

For peer review only

For peer review only - http://bmjopen.bmj.com/site/about/guidelines.xhtml

Background

Globally, 10.6 million people developed tuberculosis (TB) in 2021, of whom 3.4 million (32.1%) were women.[1] TB incidence peaks in women during the reproductive years,[2,3] is a leading cause of mortality among women of childbearing age[4] and a significant contributor to maternal mortality.[5] Pregnant women with TB are at high risk of adverse pregnancy outcomes including prematurity, low birthweight and stillbirth.[6–8]

TB is common in settings with high HIV prevalence, especially in sub-Saharan Africa. People living with HIV have a 2.5 times increased risk of developing TB despite access to antiretroviral therapy (ART),[9] and TB remains the leading cause of death among people living with HIV.[10] Pregnant women with TB and HIV are at increased risk of adverse pregnancy outcomes[11,12] and higher maternal and infant mortality.[13–16] There are limited data on TB treatment outcomes amongst pregnant women since pregnancy status is not routinely captured or reported as a TB programme indicator globally and pregnancy testing results are not routinely captured for women with TB. While ART[17] and TB preventive treatment (TPT)[18] reduces the risk of TB disease, an estimated 150,600 pregnant women and 49,000 postpartum women develop TB annually, with women in Africa accounting for 40% of this burden.[19] There is a need to integrate routine pregnancy indicators in TB programmes to improve surveillance of pregnant women with TB and improve their and their infants' outcomes.

In high TB burden settings, the WHO recommends screening pregnant women for TB at every contact with a healthcare worker.[20] In South Africa, routine testing of all pregnant women for TB and providing TPT once TB disease is excluded is recommended[18] but the extent of implementation is unknown and most likely incomplete. There is a pressing need for improved data from routine and programmatic sources to identify programmatic gaps and inform evidence-based research interventions to improve health outcomes for pregnant women with TB and their infants. This study used routine data across three health programs (TB, maternal health, and HIV services) to determine risk factors associated with an unfavourable pregnancy outcome for women with TB.

Methods

Setting

For peer review only - http://bmjopen.bmj.com/site/about/guidelines.xhtml

In 2021, the estimated TB incidence in South Africa was 513 cases per 100,000 population[1] and HIV prevalence for adults (15+ years) was 17%.[21] A third of women who received antenatal care in the public sector were living with HIV in 2019[22] and the HIV prevalence among persons with TB was 57% in 2021.[1] Maternal mortality was 139.3 deaths per 100,000 live births (2007-2015).[23] Although mortality among people with TB had declined between 2007 and 2016, approximately 12% of people with TB died in 2016.[24] TB was responsible for 11% of maternal deaths between 2014 and 2016 in South Africa.[24] Maternal mortality for the Western Cape province ranged between 43.6 and 66.8 per 100 000 live births at healthcare facilities in 2017-2019.[25]

We used routine health data from two large health sub districts (Khayelitsha and Tygerberg) in the Cape Metro, Western Cape Province, South Africa. The Cape Metro has a population of approximately 4.6 million people (two thirds of the provincial population); HIV/AIDS and TB were among the top six causes of death in 2015.[26] The antenatal HIV prevalence was 22% in 2019.[27] There were 26,000 newly diagnosed persons with TB and more than 2,000 TB deaths in 2022.[28] Khayelitsha is a peri-urban low resourced health sub district, with a mix of formal and informal housing and an estimated population of 447,000 people.[29] It is serviced by a district hospital and 10 primary healthcare (PHC) facilities.[26] Tygerberg is a large health subdistrict, with a population of 740,000 people,[29] most of whom live in formal dwellings. It has a tertiary hospital, a district level hospital and 21 PHC facilities.[26]

Study design

This was a retrospective analysis of programmatic data collected as part of a larger health system strengthening study (LINKEDin), which aimed to reduce initial loss to follow-up for people diagnosed with TB in three South African provinces.[30] This analysis included all women routinely diagnosed with TB in the Western Cape (WC) sub-districts, who were also documented to have been pregnant during their TB episode.

Study population

We included data for all women who were routinely diagnosed with TB at either a hospital or PHC in the 2 subdistricts between October 2018 and March 2020 and who had a pregnancy that overlapped with their TB episode (See Table 1 for definition of key terms),

For peer review only - http://bmjopen.bmj.com/site/about/guidelines.xhtml

as recorded in routine health records. We included pregnant women diagnosed with drug susceptible (DS) and drug-resistant (DR) TB.

Data collection

Data were extracted from the Western Cape Provincial Health Data Centre (PHDC). The PHDC is a comprehensive linked health information exchange in the province and uses unique identifiers to integrate all electronic health data from routine health information systems in the public sector (laboratory, pharmacy, administrative and other clinical data) into single patient level records to support improved patient care.[31] PHDC outputs include line lists (cascades) of patients with specific conditions e.g. TB, HIV, pregnancy.[31] These are updated daily and can be used to track patients at each healthcare visit and identify those requiring follow-up, including those newly diagnosed with TB who have not yet been entered into a TB treatment register and initiated on TB treatment.

We accessed the de-identified TB cascade from the PHDC and filtered for pregnancy status, to identify all women with TB who were pregnant during the study period in the 2 sub-districts. We used the unique identifier for these individuals to access data contained in the HIV and pregnancy cascades. Cascades are individual patient level views of specific health conditions which include key dates, evidence, outcomes, and co-morbidities relevant for patient management. We merged the three cascades and checked our dataset for consistency, confirming that all women included in our cohort had a pregnancy that overlapped with their TB episode (Table 1).

We extracted demographic (age) and clinical variables for TB, HIV, and pregnancy (e.g., *Mycobacterium tuberculosis* drug susceptibility status, HIV status, CD4 count, gravidity, infant birthweight etc) and key dates (date of TB diagnosis, date first known to the public antenatal services, date of delivery etc). To determine TB treatment outcomes, we reviewed the evidence recorded in the PHDC relating to evidence of linkage to a TB treatment facility and if linked, we extracted their TB treatment initiation and outcome data (cured, successfully completed, treatment failure, loss to follow up, died, not evaluated). We allocated a TB treatment outcome (favourable or unfavourable) as per our definition (Table 1). We used an expanded definition of unfavourable TB outcome to include newly diagnosed persons with TB who did not link to care and initiate treatment

For peer review only - http://bmjopen.bmj.com/site/about/guidelines.xhtml

at a TB treatment facility. To determine a pregnancy outcome, we reviewed live birth, infant birthweight, and gestational period and allocated a pregnancy outcome (favourable or unfavourable), as per definitions in Table 1. Women who had one or more of these variables missing, had a pregnancy outcome of unknown asssigned. Pregnancy outcomes could occur either during or after the end of the TB episode (Figure 1).

Key variable definitions

Table 1. Key variable definitions for TB, HIV, and pregnancy outcomes

**Definition of terminology**	
**TB episode**	Period from the date of TB diagnosis to the recorded date of TB outcome or death.
**TB diagnosis**	Includes a bacteriological (evidence of a positive TB laboratory test) or clinical diagnosis of TB (including evidence of TB drugs administered as part of a therapeutic regimen, or primary ICD10 codes and inclusion in a primary care TB register).
**Pregnancy episode**	The period from the first electronically captured evidence of a pregnancy documented in antenatal health services to the date of delivery or of any other pregnancy outcome.
**Overlapping pregnancy**	Date of the first evidence of the pregnancy was ≤9 months prior to the date of the TB diagnosis or ≤18 months after the date of the TB diagnosis
**TB treatment outcomes**	
Favourable TB outcome	Recorded in the routine health service data as treatment success (bacteriologically cured or successfully completed TB treatment).
Unfavourable TB outcome (expanded TB treatment outcome definition)	Recorded in the routine health service data as lost to follow up, died during treatment, treatment failed or not evaluated (WHO TB outcomes) <u>OR</u> loss to follow up prior to linkage to TB care (including died prior to linkage, i.e. "initial loss to follow-up").
**Linkage to TB care**	Evidence of accessing a TB treatment facility for TB treatment (TB hospital or a PHC facility offering TB treatment) after the diagnosis of TB.

For peer review only - http://bmjopen.bmj.com/site/about/guidelines.xhtml

**Pregnancy outcomes**	
Favourable pregnancy outcome	A pregnancy that resulted in the live birth of an infant weighing ≥2500g and with a gestation period ≥ 37 weeks.
Unfavourable pregnancy outcome	A pregnancy that resulted in either a live birth of an infant weighing <2500g and/or with a gestation period < 37 weeks or where there was a still birth, miscarriage, termination, the mother dying prior to delivery or early neonatal death.

Patient and Public involvement

As this study used data at the programme level, patients were not directly involved in any aspect of the study.

Statistical analysis

Descriptive statistics were used to describe baseline characteristics, the timing of pregnancy and delivery compared to the TB episode, stratified by HIV status. A logistic regression model was used to determine risk factors for unfavourable pregnancy outcomes including TB treatment outcomes and other predictors. After univariate analyses, predictors were added incrementally, observing the change in significance of the likelihood ratio test of each model, to produce a final adjusted model. The relationship between predictors was considered and we avoided co-linearity in the final model. SAS software (version 9.4; SAS Institute, Inc, Cary, NC) was used for analysis. -

Ethical considerations

The Health Research Ethics Committee of Stellenbosch University (N18/07/069) approved the study, which was conducted according to the guiding principles within the Declaration of Helsinki. Approvals were received from the Western Cape Department of Health and the City of Cape Town Health Directorate. We received a waiver of individual consent for this routinely collected health data.

Results

Overall

For peer review only

For peer review only - http://bmjopen.bmj.com/site/about/guidelines.xhtml

A total of 260 pregnant women were diagnosed with TB between October 2018 and March 2020, of whom 67 had a pregnancy recorded prior to their TB diagnosis. We excluded 12 women because their pregnancy outcome was also recorded prior to their TB diagnosis. Figure 1 shows pregnancy episodes in relation to their TB episodes. There were 111 women who had their pregnancy outcome documented before their TB outcome (i.e. who delivered while still on TB treatment), and 137 women who had their pregnancy outcome documented after their TB outcome (i.e. who delivered after their TB treatment).

Overall, the median age was 27.7 years (IQR: 22.7-32.3), range 14-43 years. The majority of women (191/248; 77%) had bacteriologically confirmed TB, 170/248 (69%) were treated for drug-susceptible TB and 10/248 (4%) were treated for drug-resistant TB. Among the 248 women included in analysis, all had an HIV status recorded, half (130/248; 52%) were living with HIV. Women living with HIV (WLWH) were slightly older (29.1 years; IQR 25.7-33.4) compared to HIV negative women (25.4 years; IQR 20.6-30.1). Of WLWH, the median CD4 count at TB diagnosis was 236 cells/µL(IQR:127-423) and all were on antiretroviral therapy (ART) at the time of their TB diagnosis, with 56/130 (43%) having initiated ART during their pregnancy. Supplementary Table S1 provides characteristics of pregnant women with HIV by pregnancy status at the time ART was initiated.Overall, 9/248 of women (4%) died, of whom 6/9 (66%) were living with HIV. Most women (6/9) died >60 days after their TB diagnosis had been made (Table 2).

For nearly half of the women (121/248; 49%), this was their first pregnancy. The majority (193/248; 78%) had their pregnancy recorded at or after the time their TB diagnosis was made, with most (124/193; 64%) delivering after their TB outcome was documented (Figure 1). A third (83/248; 33%) were documented to have attended antenatal services late, presenting for the first visit only within 30 days of their delivery, with most of these women (67/83; 81%) only presenting to services within 2 days of delivering their infant. Overall, the median gestation time was 40 weeks (IQR 35-40 weeks). Amongst infants overall, the median birth weight was 2875 grams (IQR:2445-3270 grams) (Table 2).

TB treatment outcomes

For peer review only - http://bmjopen.bmj.com/site/about/guidelines.xhtml

Using the more comprehensive definition including initial loss to follow-up, a third (79/248; 32%) of pregnant women had an unfavourable TB outcome; 44/130 (33.8%) among those living with HIV and 35/118 (29.7%) who were HIV negative. Of those with an unfavourable TB outcome, 34% (27/79) were never linked to TB care and were not recorded to have started TB treatment in a TB treatment register. Of those not linked to care, a quarter (7/27; 26%) died (3 HIV negative and 4 HIV positive) (Figure 2).

Pregnancy outcomes

Pregnancy outcomes were documented in 215/248 (86.7%) women. Overall, 74/215 (34.4%) women had an unfavourable pregnancy outcome; 40/106 (37.7%) among those living with HIV and 34/109 (31.2%) among HIV negative women. Overall, unfavourable pregnancy outcomes were higher among women who had unfavourable TB outcomes (32/79; 40.5%) vs. favourable TB outcomes (42/169; 24.9%), irrespective of HIV status (Figure 2).

Risk factors associated with unfavourable pregnancy outcome

Pregnant women with TB were at increased odds of having an unfavourable pregnancy outcome if they were older [adjusted odds ratio (aOR) 3.99: 95% confidence interval (CI):1.37-11.57], if they were living with HIV (aOR 2.72: 95%CI 1.2-6.18), if they had an unfavourable TB outcome (aOR 2.29: 95%CI: 1.03-5.08) and if they presented late to antenatal services (≤30 days prior to their delivery), compared to attending antenatal services for at least 121 days (aOR 10.57: 95%CI:4.01-27.89). Women were less likely to have an unfavourable pregnancy outcome with increasing gravidity (aOR 0.55: 95%CI:0.37-0.81) (Table 3).

Discussion

This analysis includes a large cohort of women diagnosed with TB in routine services and who had a pregnancy that overlapped their TB episode. Half of these women were living with HIV, higher than the estimated antenatal HIV prevalence rate of 22% in the setting.[27] All HIV-positive women were on ART; more than half (57%) were already on ART prior to their pregnancy, with the balance initiating ART during their pregnancy. Overall, these findings indicate good HIV screening and ART initiation services generally and specifically

For peer review only

among pregnant women attending public antenatal services. As comparison, approximately 54% of all people living with HIV were on ART in 2019 in Cape Town. [32]

Overall, a high rate of 34% of women with TB had an unfavourable pregnancy outcome. A systematic review of 69 studies (published 2009-2021), the majority of which were from low and middle income countries, reported poor pregnancy outcomes for women with TB, with most studies reporting low birthweight and/or preterm labour and/or spontaneous abortions.[33] A South African study reported 48% of women routinely treated for MDR/RR-TB during 2013 to 2017, had an unfavourable pregnancy outcome,[34] higher than our study, where the majority had drug-sensitive TB. Other South African studies with cohorts between 2011[15] and 2014[14] showed that of infants born to HIV-positive mothers with TB, between 20% and 70% had a gestation period <37 weeks and between 21% and 59% had a low birth weight. Since these previous studies, there have been many substantial advances including positive policy changes in our setting including universal HIV testing, universal ART rollout[35] and pregnant women being eligible for TB preventive therapy irrespective of HIV status.[18] Yet our findings show similar high rates of poor pregnancy outcomes in women, irrespective of HIV status, which is of highly concerning. Our data highlights the need for additional support and internventions required for pregnant women with TB need including strategies to improve the integration of TB and maternal services to improved TB and pregnancy outcomes for these women. There is a clear need for additional work to understand the care pathway and cascade for these women, and to better understand barriers and enablers to care and determine how they navigate TB, maternal and HIV services in parallel and the gaps between policy and implementation. We also note that comparison across studies is challenging as the definition of an unfavourable pregnancy outcome varied by study. A standardized definition including all variables that affect both maternal and infant outcomes is needed.

Having a poor TB treatment outcome was associated with having a poor pregnancy outcome, which is biologically plausible. We are not aware of any other studies that have reported on poor TB outcomes as a risk factor for poor pregnancy outcomes. A study among pregnant women with MDR/RR-TB found that 40% had an unfavourable TB treatment outcome, as per traditional WHO definitions.[7] This study also found a third of women had an unfavourable TB outcome, but used a broader definition to include those

For peer review only - http://bmjopen.bmj.com/site/about/guidelines.xhtml

lost prior to registration/treatment initiation.[36] Our study identified a high proportion of women who did not link to TB care; 43% among HIV-negative women and 27% among HIV-positive women. Essentially, these women had accessed health services, had a TB testing, yet were never registered at a TB treatment facility, i.e., were not documented to have ever started on TB treatment and were not reported nationally or to WHO. Routine TB reporting use the TB treatment register as their point of entry to determine the outcome of TB episode, thereby missing pregnant women who are lost prior to 'linkage' ("initial loss to follow-up) and who are not registered and notified as a person with TB. This group may represent a large source of mortality and is a clear target group for inteventions to improve linkage to care and retention in care.

While initial loss to follow up is not currently reported as a routine indicator in the TB programme, it is estimated that approximately 12% among drug-sensitive[37] and 37% among drug-resistant[38] TB patients are never started on treatment, highlighting a critical need to improve linkage to care and registration at a TB treatment facility. Pregnant women diagnosed with TB are a highly vulnerable group who require more intensive follow up to ensure linkage to care and should be prioritized for TB care to ensure better TB and pregnancy outcomes, and to reduce mortality. Routine antenatal and postnatal services should serve as an important trigger to link women to TB treatment following screening and testing. Being lost to the health system after a TB diagnosis may be the underlining reason for having an unfavourable pregnancy outcome. The association between TB and pregnancy outcomes should be further explored to better understand how these are associated. The lack of recording these women in TB treatment registers means that their data is not included in routine recording and reporting, further excerbating the gaps in surveillance of TB in women of childbearing age.

Most women had their pregnancy recorded only after they were diagnosed with TB. Offering effective contraception to women at TB treatment initiation[33,39] and screening for pregnancy at each TB-related visit, with integration of reproductive health services as part of TB care is critically important.[40] South African policy includes the provision of long-acting contraceptive injectables for women with TB. In addition to counselling on contraceptive services, women should be made aware of their increased risk of poorer pregnancy outcomes and the need for additional care if they conceive during their TB

For peer review only - http://bmjopen.bmj.com/site/about/guidelines.xhtml

episode. If adequate counselling and assessment of family planning at the start of TB treatment is being implemented as per local guidelines, and women continue to fall pregnant during their TB episode, then there could be other factors associated with this. Socio-behavioural research may elicit such factors and provide more person-centered understanding that could guide better implementation of exitsing or more patient-centered policies .

Living with HIV was found to be a risk factor for having an unfavourable pregnancy outcome, similar to previous studies. However, in our study, women had similar poor pregnancy outcomes irrespective of whether ART was started prior to or during pregnancy. A systematic review, including 73 cohort studies found that ART reduced the risk of adverse perinatal outcomes in pregnant women living with HIV, but the risk remained higher than in HIV-negative women.[41] South African studies have shown that although increased ART coverage improved birth outcomes, HIV-positive women were significantly more likely to have a stillbirth or a gestation period < 37 weeks compared to HIV-negative women[42] and were at increased risk of having a 'small for gestational age' infant, regardless of when they had initiated ART.[43] While pregnancy remains an excellent opportunity to start ART, the WHO recommends the immediate initiation of lifelong ART for all people living with HIV.[44] Despite a universal test and treat policy for HIV, the findings from our study shows that many women start ART only at antenatal presentation. A significant improvement in community-based HIV testing and treatment to support earlier ART initiation is essential. ART initiation prior to pregnancy may reduce adverse pregnancy outcomes for these women and also TB treatment outcomes.

Our findings show that a third of women (25% of women living with HIV and 42% of HIV-negative women) were only documented to have sought care at public antenatal services during the month prior to delivery, highlighting the challenge of 'late booking'.All antenatal, TB and HIV services are free of direct charge in South Africa in the public sector. This proportion is higher than in the 2019 South African Antenatal survey, where only a small proportion of women (5.6%) attended their first antenatal visit in the last trimester of their pregnancy.[22] In the Cape Town Metro, 75% of women attend antenatal services before 20 weeks.[27] South Africa guidelines indicate that booking should be as early as possible [45] to ensure optimal antenatal care. Clearly, pregnant women with TB are an

For peer review only - http://bmjopen.bmj.com/site/about/guidelines.xhtml

especially high-risk group overall. Those who book late should be tested for TB as a priority and offered TPT. Tracking pregnancy using 'action lists' may offer the opportunity for personnel in the TB programme to create awareness around the importance of adequate antenatal visits and urge these women to access maternal services earlier in their pregnancy.

We found that gravidity had a protective effect. This could be an example of the "healthy mother effect," where women who have had successful pregnancies in the past are more likely to have successful pregnancies in the future.[46] This finding should be interpreted with caution.

Our study addresses a key yet neglected aspect of TB and maternal child health, using routine health data from multiple integrated sources that allowed us to evaluate TB and pregnancy outcomes with consideration of HIV. We demonstrate poor pregnancy outcomes in pregnant women with TB, half of whom were also living with HIV. Using the PHDC strengthened our ability to determine more inclusive definitions of favourable and unfavourable TB outcomes and enabled us to include all pregnant women diagnosed with TB, so we could determine both pre- and post-loss to follow up during TB care. Using our expanded definition of an 'unfavourable' TB outcome, this study was able to reflect more accurately 'linkage to care' within the TB program for pregnant women. From a program perspective, having a maternal cascade is a benefit for the monitoring of the maternal and infant health program in services. Health services should allow for integrated tools to provide optimal care for patients across multiple programs: maternal, TB and HIV programs.

We could not determine a pregnancy outcome for 13% of our cohort, due to missing data. This may be due to women delivering outside the province, data linkage limitations between mother and infant, or incomplete data capture, potentially resulting in a biased outcome and over-reporting of unfavourable pregnancy outcomes. Missing data is a limitation when using routine health data sources. A second limitation is that while the multivariable regression model was able to control for selected factors, residual confounding could have occurred due to other maternal factors (gestational diabetes, hypertension, anaemia) or socio-economic factors (poverty, substance use, educational

For peer review only - http://bmjopen.bmj.com/site/about/guidelines.xhtml

status). These data were not available for analysis and should be included in future work. A further limitation is the inability to explain the time between presenting with TB and the timing of pregnancy.

Conclusions

TB care needs to be strengthened for pregnant women and health services need to think through how best to optimise maternal services within the TB program. We used routine data to track women across the TB, HIV and maternal healthcare cascades, allowing for more inclusive definitions and analysis of linkage to care and TB and pregnancy outcomes. Of major concern and a new finding is that a high proportion of women diagnosed with TB never started TB treatment. Pregnant women with TB are a highly vulnerable group who need additional support prior to, during and after TB treatment to reduce poor pregnancy outcomes, even those who are HIV-negative. Going forward, recording of pregnancy status should be a key indicator piloted as part of TB treatment registries, as this would result in better quality data and offer opportunites for earlier intervention and support of pregnant and postpartum women with TB.

For peer review only

For peer review only - http://bmjopen.bmj.com/site/about/guidelines.xhtml

References

1. World Heath Organization. Global tuberculosis report 2022 [Internet]. Geneva; Available from: https://www.who.int/teams/global-tuberculosis-programme/tb-reports/global-tuberculosis-report-2022
2. Deluca A, Chaisson R, Martinson N. Intensified case finding for tuberculosis in prevention of mother-to-child transmission programs: A simple and potentially vital addition for maternal and child health. J Acquir Immune Defic Syndr. 2009;50(2):196–9.
3. Gupta A, Nayak U, Ram M, Bhosale R, Patil S, Basavraj A, et al. Postpartum tuberculosis incidence and mortality among HIV-infected women and their infants in Pune, India, 2002-2005. Clin Infect Dis. 2007;45(2):241–9.
4. Zumla A, Bates M, Mwaba P. The neglected global burden of tuberculosis in pregnancy. Lancet Glob Heal [Internet]. 2014;2(12):e675–6. Available from: http://dx.doi.org/10.1016/S2214-109X(14)70338-9
5. Loto OM, Awowole I. Tuberculosis in pregnancy: A review. J Pregnancy. 2012;2012(379271).
6. Alene KA, Murray MB, van de Water BJ, Becerra MC, Atalell KA, Nicol MP, et al. Treatment Outcomes Among Pregnant Patients With Multidrug-Resistant Tuberculosis. JAMA Netw Open. 2022;5(6):e2216527.
7. Mokhele I, Jinga N, Berhanu R, Dlamini T, Long L, Evans D. Treatment and pregnancy outcomes of pregnant women exposed to second-line anti-tuberculosis drugs in South Africa. BMC Pregnancy Childbirth [Internet]. 2021;21(1):1–11. Available from: https://doi.org/10.1186/s12884-021-03956-6
8. Jana N, Barik S, Arora N, Singh AK. Tuberculosis in pregnancy: The challenges for South Asian countries. J Obstet Gynaecol Res. 2012;38(9):1125–36.
9. Yaghoubi A, Salehabadi S, Abdeahad H, Hasanian SM, Avan A, Yousefi M, et al. Tuberculosis, human immunodeficiency viruses and TB/HIV co-infection in pregnant women: A meta-analysis. Clin Epidemiol Glob Heal. 2020;8(4):1312–20.
10. Zumla A, Malon P, Henderson J, Grange JM. Impact of HIV infection on tuberculosis. Postgr Med J. 2000;76:259–68.
11. Rollins NC, Coovadia HM, Bland RM, Coutsoudis A, Bennish ML, Patel D, et al. Pregnancy outcomes in HIV-infected and uninfected women in rural and urban South Africa. J Acquir Immune Defic Syndr. 2007;44(3):321–8.

For peer review only

For peer review only - http://bmjopen.bmj.com/site/about/guidelines.xhtml

12. Arab K, Spence AR, Czuzoj-Shulman N, Abenhaim HA. Pregnancy outcomes in HIV-positive women: a retrospective cohort study. Arch Gynecol Obstet. 2017;295(3):599–606.

13. Calvert C, Ronsmans C. The contribution of HIV to pregnancy-related mortality: A systematic review and meta-analysis. Aids. 2013;27(10):1631–9.

14. Salazar-Austin N, Hoffmann J, Cohn S, Mashabela F, Waja Z, Lala S, et al. Poor Obstetric and Infant Outcomes in Human Immunodeficiency Virus-Infected Pregnant Women with Tuberculosis in South Africa: The Tshepiso Study. Clin Infect Dis. 2018;66(6):921–9.

15. Bekker A, Schaaf HS, Draper HR, Kriel M, Hesseling AC. Tuberculosis disease during pregnancy and treatment outcomes in HIV-infected and uninfected women at a referral Hospital in Cape Town. PLoS One. 2016;11(11):1–14.

16. Mnyani CN, McIntyre JA. Tuberculosis in pregnancy. BJOG An Int J Obstet Gynaecol. 2011;118(2):226–31.

17. Lawn SD, Harries AD, Williams BG, Chaisson RE, Losina E, Cock KM De, et al. Antiretroviral therapy and the control of HIV-associated tuberculosis. Will ART do it? Int J Tuberc Lung Dis. 2011;15(5):571–81.

18. South African National Department of Health. National Guidelines on the Treatment of Tuberculosis Infection [Internet]. 2023. Available from: https://www.knowledgehub.org.za/system/files/elibdownloads/2023-03/Health_Latent TB Infection_2023_web.pdf

19. Mafirakureva N. Revisiting the burden of TB in pregnant and post-partum women. In: International Journal of Tuberculosis and Lung Disease Abstract book 50th World Conference Hyderabad, India [Internet]. 2019. Available from: https://theunion.org/our-work/conferences/history-of-the-union-world-conference-on-lung-health/conference-abstract-books

20. World Health Oghanization. WHO consolidated guidelines on tuberculosis Module 2: Screening Systemic screening for tuberculosis disease [Internet]. World Health Organization. 2020. Available from: https://apps.who.int/iris/handle/10665/340255

21. UNAIDS:https://aidsinfo.unaids.org/. Aids Information [Internet]. 2020. [cited 2020 May 11]. Available from: https://aidsinfo.unaids.org

22. Woldesenbet, S.A., Lombard, C., Manda, S., Kufa, T., Ayalew, K., Cheyip M., and Puren A. The 2019 National Antenatal Sentinel HIV Survey, South Africa, National

For peer review only - http://bmjopen.bmj.com/site/about/guidelines.xhtml

Department of Health [Internet]. 2021. Available from: https://www.nicd.ac.za/wp-content/uploads/2021/11/Antenatal-survey-2019-report_FINAL_27April21.pdf

23. Bomela NJ. Maternal mortality by socio-demographic characteristics and cause of death in South Africa: 2007-2015. BMC Public Health. 2020;20(1):1–20.

24. Loveday M, Mzobe YN, Pillay Y, Barron P. Figures of the dead: A decade of tuberculosis mortality registrations in South Africa. South African Med J. 2019;109(10):728–32.

25. Kalk E, Heekes A, Slogrove AL, Phelanyane F, Davies MA, Myer L, et al. Cohort profile: the Western Cape Pregnancy Exposure Registry (WCPER). BMJ Open. 2022;12(6).

26. Western Cape Government: Health. Cape Metro Health District Plan (2021/22 to 2023/24). 2021.

27. Woldesenbet, S.A., Lombard, C., Manda, S., Kufa, T., Ayalew, K., Cheyip M., and Puren A. The 2019 National Antenatal Sentinel HIV Survey, South Africa, National Department of Health. 2021.

28. Western Cape Government Department of Health. Western Cape Provincial Tuberculosis (TB) Dashboard [Internet]. 2022 [cited 2022 Oct 15]. Available from: https://www.westerncape.gov.za/site-page/provincial-tb-dashboard

29. Western Cape Government Health Department. Circular H 102/2020 Covid-19 Population Data. Chief Dir Strateg Heal Support [Internet]. 2020;1–123. Available from: https://www.westerncape.gov.za/assets/departments/health/h_102_2020_covid-19_population_data.pdf

30. Osman M, Meehan S-A, Delft A von, Boulle A, Dunbar R, Smith M, et al. Reducing initial loss to follow-up among people with TB in Cape Town, South Africa. In: 50th World Conference on Lung health International Union Against Tuberculosis and Lung Disease (The Union) Abstract Book [Internet]. 2021. Available from: https://repositorio.conacyt.gov.py/handle/20.500.14066/3521

31. Boulle A, Heekes A, Tiffin N, Smith M, Mutemaringa T, Zinyakatira, N, Phelanyane F, et al. Data Centre Profile: The Provincial Health Data Centre of the Western Cape. Int J Popul Data Sci. 2019;0(October).

32. Western Cape Government Department of Health. Cape Metro District Health Plan 2018/19 -2020/21 [Internet]. 2018. Available from: http://www.health.gov.za/DHP/docs/DHP2018-

For peer review only - http://bmjopen.bmj.com/site/about/guidelines.xhtml

21/Western_Cape/Metro_District_Health_Plan.pdf

33. Nguyen Y, McNabb KC, Farley JE, Warren N. Examining family planning and adverse pregnancy outcomes for women with active tuberculosis disease: a systematic review. BMJ Open. 2022;12(3):e054833.

34. Loveday M, Hughes J, Sunkari B, Master I, Hlangu S, Reddy T, et al. Maternal and Infant Outcomes among Pregnant Women Treated for Multidrug/Rifampicin-Resistant Tuberculosis in South Africa. Clin Infect Dis. 2021;72(7):1158–68.

35. South African National Department of Health. Circular: Implementation of the universal test and treat strategy for HIV positive patients and differentiated care for stable patients [Internet]. 2016. Available from: https://sahivsoc.org/Files/22 8 16 Circular UTT Decongestion CCMT Directorate.pdf

36. Linh NN, Viney K, Gegia M, Falzon D, Glaziou P, Floyd K, et al. World Health Organization treatment outcome definitions for tuberculosis: 2021 update. Eur Respir J [Internet]. 2021;58(2). Available from: http://dx.doi.org/10.1183/13993003.00804-2021

37. Naidoo P, Theron G, Rangaka MX, Chihota VN, Vaughan L, Brey ZO, et al. The South African Tuberculosis Care Cascade: Estimated Losses and Methodological Challenges. J Infect Dis. 2017;216(Suppl 7):S702–13.

38. Cox H, Dickson-Hall L, Ndjeka N, van't Hoog A, Grant A, Cobelens F, et al. Delays and loss to follow-up before treatment of drug-resistant tuberculosis following implementation of Xpert MTB/RIF in South Africa: A retrospective cohort study. PLoS Med. 2017;14(2):1–19.

39. Schnippel K, Ndjeka N, Conradie F, Berhanu R, Claasen Z, Banoo S, et al. A call to action: Addressing the reproductive health needs of women with drug-resistant tuberculosis. South African Med J. 2016;106(4):333–4.

40. Maugans C, Loveday M, Hlangu S, Waitt C, Schalkwyk M Van, Water B Van De, et al. Best practices for the care of pregnant people living with TB. Int J Lung Dis. 2023;27(January):357–66.

41. Portwood C, Sexton H, Kumarendran M, Brandon Z, Kirtley S, Hemelaar J. Adverse perinatal outcomes associated with antiretroviral therapy in women living with HIV: A systematic review and meta-analysis. Front Med. 2023;9(February).

42. Cornish EF, Hudson J, Sayers R, Loveday M. Improving access to contraception through integration of family planning services into a multidrug-resistant tuberculosis

For peer review only - http://bmjopen.bmj.com/site/about/guidelines.xhtml

treatment programme. BMJ Sex Reprod Heal. 2020;46(2):152–5.

43. Malaba TR, Mukonda E, Matjila M, Madlala HP, Myer L, Newell ML. Pregnancy outcomes in women living with HIV and HIV-negative women in South Africa: Cohort analysis based on bias-corrected gestational age. Paediatr Perinat Epidemiol. 2022;36(4):525–35.

44. World Heath Organization. Consolidated Guidelines on the use of Antiretroviral drugs for treating and preventing HIV infection. Recommendations for a public health approach. 2nd Edition. 2016.

45. South African National Department of Health. Guidelines for maternity care in South Africa. A manual for clinics, community health centres and district hospitals. Fourth Edition. [Internet]. 2016. Available from: https://knowledgehub.health.gov.za/elibrary/guidelines-maternity-care-south-africa-2016

46. Whitley E, Doyle P, Roman E, De Stavola B. The effect of reproductive history on future pregnancy outcomes. Hum Reprod. 1999;14(11):2863–7.

For peer review only

For peer review only - http://bmjopen.bmj.com/site/about/guidelines.xhtml

Acknowledgements

We wish to acknowledge our implementing partners; the University of Cape Town and the Centre for Infectious Disease Epidemiology and Research (CIDER) in the Western Cape Province. We further acknowledge the staff at the Western Cape Provincial Health Data Centre (PHDC) for their invaluable assistance. We highly appreciate input from the health staff at the provincial, district and sub-district health offices and those at the facilities in which the study was implemented.

Funding

This research study and publication was supported by the Bill and Melinda Gates Foundation (BMGF), Investment INV- 007130. The contents are the responsibility of the authors and do not necessarily reflect the views of the BMGF. The funders had no role in study design, data collection and analysis, decision to publish, or preparation of the manuscript.

ACH is funded by the South African National Research Foundation SaRchi Chair in Paediatric Tuberculosis.

Conflict of Interest

The authors have no conflict of interest to declare.

Author contributions

SM and MO designed the study. SM, MO, AvD, RD, FP and MS were involved in data collection, extraction, and validation.

SM and MO produced the first draft of the manuscript. All authors reviewed the manuscript and provided critical input. All authors have reviewed the final version of the manuscript and approve of its content and submission for publication.

For peer review only

Table 2. Demographic and clinical characteristics among pregnant women with tuberculosis, by HIV status (n=248).

	Total N (%)	HIV status n (%)	
	(N=248)	HIV+ (n=130)	HIV- (n=118)
**Age in years, median (IQR)**	27.7 (22.7-32.3)	29.1 (25.7-33.4)	25.4 (20.6-30.1)
14-17 years	10 (4.1)	3 (2.3)	7 (5.7)
18-24 years	75 (30.2)	25 (19.2)	50 (40.7)
25-34 years	129 (52.0)	82 (63.1)	47 (38.2)
35-44 years	34 (13.7)	20 (15.4)	14 (11.4)
**Pregnancy recorded in relation to TB diagnosis**			
Pregnant before TB diagnosed	55 (22.2)	33 (25.4)	22 (18.6)
Pregnant after TB diagnosed	193 (77.8)	97 (74.6)	96 (81.4)
**TB diagnosis (%)**			
Bacteriologically confirmed	191 (77.0)	93 (71.5)	98 (83.1)
Clinically diagnosed	57 (23.0)	37 (28.5)	20 (16.9)
**Place of TB diagnosis (%)**			
Hospital	78 (31.5)	46 (35.4)	32 (27.1)
PHC facility	170 (68.5)	84 (64.6)	86 (72.9)
**TB Treatment Category**			
New	162 (65.3)	85 (65.4)	77 (65.3)
Retreatment	66 (26.6)	37 (28.5)	29 (24.6)
Unknown	20 (8.1)	8 (6.2)	12 (10.2)
**Site of disease**			
Extra-pulmonary TB	44 (17.7)	32 (24.6)	12 (10.2)
Pulmonary TB	196 (79.0)	92 (70.8)	104 (88.1)
Not specified	8 (3.2)	6 (4.6)	2 (1.7)
**Drug susceptibility status**			
DR TB	10 (4.0)	5 (3.8)	5 (4.2)
DS TB	170 (68.5)	81 (62.3)	89 (75.4)
DST not recorded	68 (27.4)	44 (33.8)	24 (20.3)
**TB outcomes (%)**			
Favourable	169 (68.1)	86 (66.2)	83 (70.3)
Unfavourable	79 (31.9)	44 (33.8)	35 (29.7)
**Linked to TB Care**			
Linkage ≤30 days	202(81.5)	107 (82.3)	95 (80.5)
Linkage >30days	19 (7.7)	11 (8.5)	8 (6.8)
Unlinked	27 (10.9)	12 (9.2)	15 (12.7)
**Died**			
Yes	9 (3.6)	6 (4.6)	3 (2.5)
No	239 (96.4)	124 (95.4)	115 (97.5)
**Time to maternal death (from date of TB diagnosis)**			
0-30 days	3 (33.3)	1 (16.7)	2 (66.7)
30-59 days	0 (0)	0 (0)	0 (0)
61-90 days	2 (22.2)	2 (33.3)	0 (0)
≥90 days	4 (44.4)	3 (50.0)	1 (33.3)

For peer review only - http://bmjopen.bmj.com/site/about/guidelines.xhtml

**Parity**				
	0	121 (48.8)	55 (42.3)	66 (55.9)
	1	71 (28.6)	47 (36.2)	24 (20.3)
	2	35 (14.1)	19 (14.63.8)	16 (13.6)
	>2	21 (8.5)	9 (6.9)	12 (10.2)
**Gravidity**				
	1	116 (46.8)	52 (40.0)	64 (54.2)
	2	71 (28.6)	46 (35.4)	25 (21.2)
	3	39 (15.7)	21 (16.2)	18 (15.3)
	>3	22 (8.9)	11 (8.5)	11 (9.3)
**Gestation (weeks), median (IQR)**		40 (35-40)	40 (35-40)	40 (35-40)
**Gestation (weeks)**				
	<30	14 (5.6)	8 (6.2)	6 (5.1)
	30-40	228 (91.9)	119 (91.5)	109 (92.4)
	>40	5 (2.0)	3 (2.3)	2 (1.7)
	Not recorded	1 (0.4)	0 (0)	1 (0.8)
**Birth Weight, in grams, median (IQR)**		2870 (2445-3270)	2835 (2400-3320)	2902 (2500-3240)
**Birth Weight (g)**				
	very low/low (500-2499)	56 (22.6)	31 (23.8)	25 (21.2)
	normal (2500-3900)	142 (57.3)	66 (50.8)	76 (64.4)
	>3900	4 (1.6)	2 (1.5)	2 (1.7)
	unknown	46 (18.5)	31 (23.8)	15 (12.7)
**Time known to ANC services (days) (%)**				
	0-30	83 (33.5)	33 (25.4)	50 (42.4)
	31-120	70 (28.2)	41 (31.5)	29 (24.6)
	≥ 121	95 (38.3)	56 (43.1)	39 (33.1)
**Pregnancy Outcome (%)**				
	Favourable	141 (56.9)	66 (50.8)	75 (63.6)
	Unfavourable	74 (29.8)	40 (30.8)	34 (28.8)
	Unknown*	33 (13.3)	24 (18.5)	9 (7.6)

ANC: antenatal care, DR: drug-resistant; DS: drug-susceptible; DST: drug-susceptible test; PHC: primary health care

*Excluded from regression model used to predict unfavourable pregnancy outcomes, as there was missing data for one or more of the following variables; live birth status, birth weight or gestational age.

For peer review only - http://bmjopen.bmj.com/site/about/guidelines.xhtml

Table 3: Multivariable logistic regression model for predictors of unfavourable pregnancy outcomes among women with tuberculosis (n = 215).

		Total	Unfavourable	%	OR (95%CI)	aOR (95%CI)
Age category	14-17 years	8	2	25.0%	0.76 (0.15-3.96)	0.5 (0.05-5.24)
	18-24 years	64	21	32.8%	1.12 (0.58-2.15)	1.56 (0.65-3.76)
	25-34 years	115	35	30.4%	reference	
	35-44 years	28	16	57.1%	3.05 (1.31-7.11)	3.99 (1.37-11.57)
HIV status	HIV+	106	40	37.7%	1.34 (0.76-2.35)	2.72 (1.2-6.18)
	HIV-	109	34	31.2%	reference	
Location of diagnosis	Hospital	64	32	50.0%	2.6 (1.42-4.76)	2.14 (0.99-4.63)
	PHC	151	42	27.8%	reference	
Pervious TB treatment history	Retreatment	55	20	36.4%	1.19 (0.62-2.29)	0.7 (0.31-1.57)
	New	142	46	32.4%	reference	
Diagnostic method (a)	Bacteriologically confirmed	168	64	38.1%	2.28 (1.06-4.89)	2.33 (0.86-6.29)
	Clinically diagnosed	47	10	21.3	reference	
Site of disease (a)	PTB	174	59	33.9%	reference	
	EPTB	37	14	37.8%	1.19 (0.57-2.47)	
DST (a)	DR TB	8	4	50.0%	1.7 (0.41-7.05)	
	DST not recorded	56	14	25.0%	0.57 (0.28-1.13)	
	DS TB	151	56	37.1%	reference	
TB treatment linkage (b)	Linked within 30 days	172	52	30.2%	reference	
	Linked after 30 days	19	6	31.6%	1.07 (0.38-2.96)	
	Never linked	24	16	66.7%	4.62 (1.86-11.45)	
TB treatment outcome (b)	Unfavourable	68	32	47.1%	2.22 (1.23-4.03)	2.29 (1.03-5.08)
	Favourable	147	42	28.6%	reference	
Time attending antenatal services	0-30 days	71	36	50.7%	4.87 (2.36-10.06)	10.57 (4.01-27.89)
	31-120 days	58	23	39.7%	3.11 (1.45-6.69)	3.54 (1.39-8.98)
	≥121 days	86	15	17.4%	reference	
Parity (c)	continuous				0.7 (0.52-0.95)	

For peer review only - http://bmjopen.bmj.com/site/about/guidelines.xhtml

Gravidity (c)	continuous				0.7 (0.53-0.94)	0.55 (0.37-0.81)

(a), (b), and (c) not added simultaneously to final model to avoid collinearity

For peer review only

For peer review only - http://bmjopen.bmj.com/site/about/guidelines.xhtml

Figure 1: Timing of pregnancy in relation to the tuberculosis episode

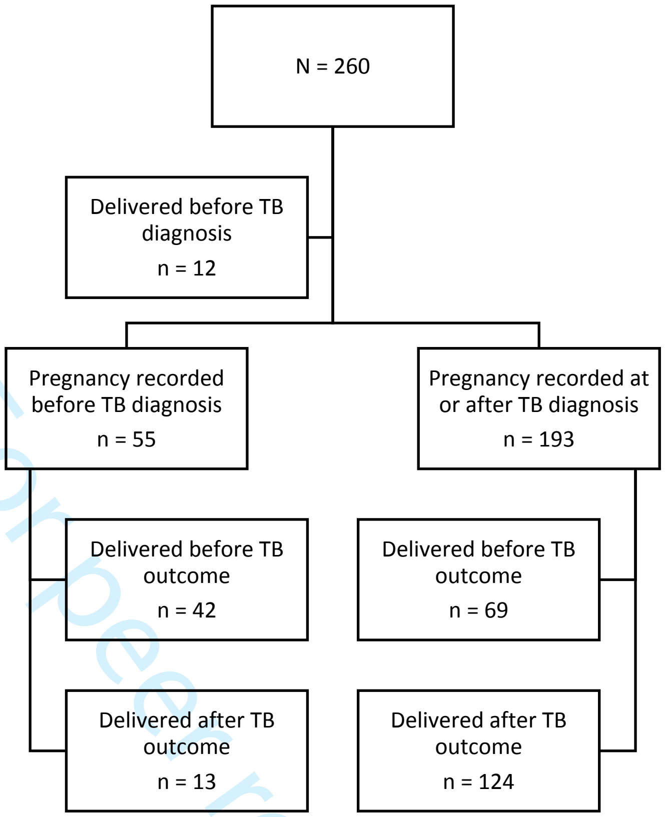

For peer review only - http://bmjopen.bmj.com/site/about/guidelines.xhtml

Figure 2: Tuberculosis outcomes and pregnancy outcomes by HIV status (n=248)

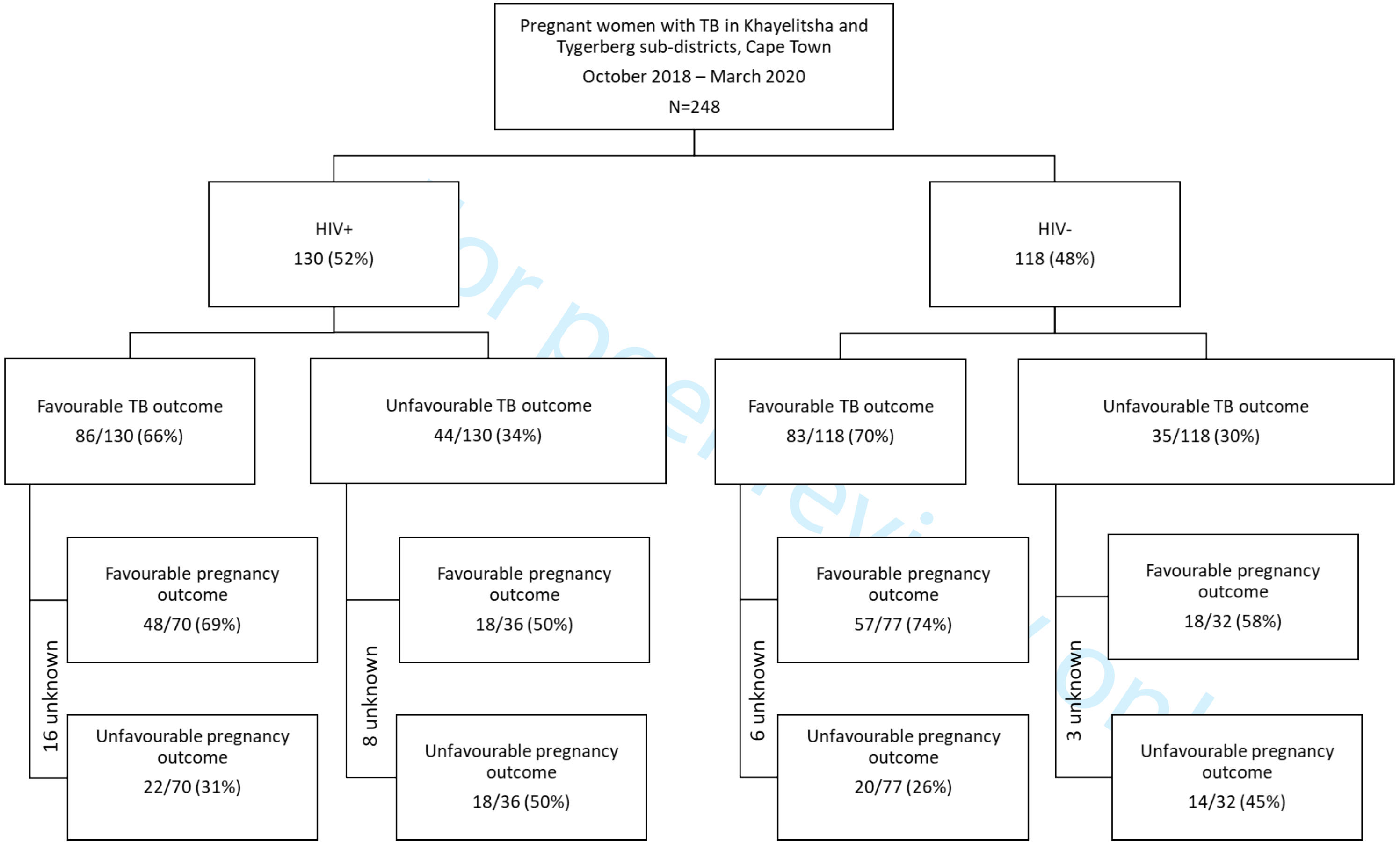

For peer review only - http://bmjopen.bmj.com/site/about/guidelines.xhtml

Supplementary Table S1: Demographic and clinical characteristics among pregnant women with tuberculosis and HIV, stratified by pregnancy status at the time antiretroviral treatment was initiated (n=130).

	Total N (%)	Pregnant at ART start n (%)	
	**(N=130)**	**Yes (n=56), 43%**	**No (n=74), 57%**
**CD4 count*, median (IQR)**	235 (127-423)	231 (100-352)	260 (134-468)
**Age**			
14-17 years	3 (2.3)	0 (0)	3 (4.1)
18-24 years	25 (19.2)	9 (16.1)	16 (21.6)
25-34 years	82 (63.1)	39 (69.6)	43 (58.1)
35-44 years	20 (15.4)	8 (14.3)	12 (16.2)
**Pregnancy recorded in relation to TB diagnosis**			
Pregnant before TB diagnosed	33 (25.4)	14 (25.0)	19 (25.7)
Pregnant after TB diagnosed	97 (74.6)	42 (75.0)	55 (74.3)
**TB treatment at ART start**			
yes	37 (28.5)	21 (37.5)	16 (21.6)
no	93 (71.5)	35 (62.5)	58 (78.4)
**Viral suppression ever**			
yes	115 (88.5)	48 (85.7)	67 (90.5)
no	15 (11.5)	8 (14.3)	7 (9.5)
**Last VL suppressed**			
yes	97 (74.6)	42 (75.0)	55 (74.3)
no	33 (25.4)	14 (25.0)	19 (25.7)
**Time known to ANC services (days) (%)**			
0-30	33 (25.4)	17 (30.4)	16 (21.6)
31-120	41 (31.5)	19 (33.9)	22 (29.7)
≥ 121	56 (43.1)	20 (35.7)	36 (48.6)
**Pregnancy Outcome (%)**			
favourable	66 (50.8)	30 (53.6)	36 (48.6)
unfavourable	40 (30.8)	17 (30.4)	23 (31.1)
unknown	24 (18.5)	9 (16.1)	15 (20.3)

*CD4 count closest to date of TB diagnosis

ART: antiretroviral treatment, VL: viral load, ANC: antenatal care

For peer review only

Pregnant women with tuberculosis have high rates of unfavourable pregnancy outcomes irrespective of HIV status in Cape Town, South Africa

Authors

Sue-Ann Meehan[1*], Anneke C. Hesseling[1], Arne von Delft[2,3], Florian M. Marx[1,4], Jennifer A. Hughes[1], Peter Bock[1], Aduragbemi Banke-Thomas[5,6], Rory Dunbar[1], Florence Phelanyane[2,3], Mariette Smith[2,3], Muhammad Osman[1,5]

*Corresponding author

Email address: sueannm@sun.ac.za (SM)

Affiliations:

[1]Desmond Tutu TB Centre, Department of Paediatrics and Child Health, Faculty of Medicine and Health Sciences, Stellenbosch University

[2]Centre for Infectious Disease Epidemiology and Research (CIDER), School of Public Health and Family Medicine, Faculty of Health Sciences, University of Cape Town

[3]Health Intelligence Directorate, Western Cape Government, Department of Health and Wellness

[4]Division of Infectious Disease and Tropical Medicine, Centre for Infectious Diseases, Heidelberg University Hospital, Heidelberg, Germany

[5]School of Human Sciences, Faculty of Education, Health and Human Sciences, University of Greenwich, London, United Kingdom

[6]Faculty of Epidemiology and Population Health, London School of Hygiene and Tropical Medicine, London, United Kingdom

For peer review only - http://bmjopen.bmj.com/site/about/guidelines.xhtml

Supplementary Table S1: Demographic and clinical characteristics among pregnant women with tuberculosis and HIV, stratified by pregnancy status at the time antiretroviral treatment was initiated (n=130).

	Total N (%)	Pregnant at ART start n (%)	
	**(N=130)**	**Yes (n=56), 43%**	**No (n=74), 57%**
**CD4 count*, median (IQR)**	235 (127-423)	231 (100-352)	260 (134-468)
**Age**			
14-17 years	3 (2.3)	0 (0)	3 (4.1)
18-24 years	25 (19.2)	9 (16.1)	16 (21.6)
25-34 years	82 (63.1)	39 (69.6)	43 (58.1)
35-44 years	20 (15.4)	8 (14.3)	12 (16.2)
**Pregnancy recorded in relation to TB diagnosis**			
Pregnant before TB diagnosed	33 (25.4)	14 (25.0)	19 (25.7)
Pregnant after TB diagnosed	97 (74.6)	42 (75.0)	55 (74.3)
**TB treatment at ART start**			
yes	37 (28.5)	21 (37.5)	16 (21.6)
no	93 (71.5)	35 (62.5)	58 (78.4)
**Viral suppression ever**			
yes	115 (88.5)	48 (85.7)	67 (90.5)
no	15 (11.5)	8 (14.3)	7 (9.5)
**Last VL suppressed**			
yes	97 (74.6)	42 (75.0)	55 (74.3)
no	33 (25.4)	14 (25.0)	19 (25.7)
**Time known to ANC services (days) (%)**			
0-30	33 (25.4)	17 (30.4)	16 (21.6)
31-120	41 (31.5)	19 (33.9)	22 (29.7)
≥ 121	56 (43.1)	20 (35.7)	36 (48.6)
**Pregnancy Outcome (%)**			
favourable	66 (50.8)	30 (53.6)	36 (48.6)
unfavourable	40 (30.8)	17 (30.4)	23 (31.1)
unknown	24 (18.5)	9 (16.1)	15 (20.3)

*CD4 count closest to date of TB diagnosis

ART: antiretroviral treatment, VL: viral load, ANC: antenatal care

For peer review only - http://bmjopen.bmj.com/site/about/guidelines.xhtml

STROBE Statement—Checklist of items that should be included in reports of ***cohort studies***

	Item No	Recommendation	Page No
**Title and abstract**	1	(*a*) Indicate the study's design with a commonly used term in the title or the abstract	2
		(*b*) Provide in the abstract an informative and balanced summary of what was done and what was found	2
**Introduction**			
Background/rationale	2	Explain the scientific background and rationale for the investigation being reported	4
Objectives	3	State specific objectives, including any prespecified hypotheses	4
**Methods**			
Study design	4	Present key elements of study design early in the paper	5
Setting	5	Describe the setting, locations, and relevant dates, including periods of recruitment, exposure, follow-up, and data collection	5
Participants	6	(*a*) Give the eligibility criteria, and the sources and methods of selection of participants. Describe methods of follow-up	5
		(*b*) For matched studies, give matching criteria and number of exposed and unexposed	N/A
Variables	7	Clearly define all outcomes, exposures, predictors, potential confounders, and effect modifiers. Give diagnostic criteria, if applicable	6 and Table 1
Data sources/ measurement	8*	For each variable of interest, give sources of data and details of methods of assessment (measurement). Describe comparability of assessment methods if there is more than one group	6
Bias	9	Describe any efforts to address potential sources of bias	6-7
Study size	10	Explain how the study size was arrived at	5
Quantitative variables	11	Explain how quantitative variables were handled in the analyses. If applicable, describe which groupings were chosen and why	6
Statistical methods	12	(*a*) Describe all statistical methods, including those used to control for confounding	7
		(*b*) Describe any methods used to examine subgroups and interactions	N/A
		(*c*) Explain how missing data were addressed	7
		(*d*) If applicable, explain how loss to follow-up was addressed	N/A
		(*e*) Describe any sensitivity analyses	N/A
**Results**			
Participants	13*	(a) Report numbers of individuals at each stage of study—eg numbers potentially eligible, examined for eligibility, confirmed eligible, included in the study, completing follow-up, and analysed	8
		(b) Give reasons for non-participation at each stage	N/A
		(c) Consider use of a flow diagram	26/27
Descriptive data	14*	(a) Give characteristics of study participants (eg demographic, clinical, social) and information on exposures and potential confounders	8-9
		(b) Indicate number of participants with missing data for each variable of interest	10
		(c) Summarise follow-up time (eg, average and total amount)	N/A
Outcome data	15*	Report numbers of outcome events or summary measures over time	9-10

For peer review only

Main results	16	(*a*) Give unadjusted estimates and, if applicable, confounder-adjusted estimates and their precision (eg, 95% confidence interval). Make clear which confounders were adjusted for and why they were included	10
		(*b*) Report category boundaries when continuous variables were categorized	8-10
		(*c*) If relevant, consider translating estimates of relative risk into absolute risk for a meaningful time period	N/A
Other analyses	17	Report other analyses done—eg analyses of subgroups and interactions, and sensitivity analyses	N/A
**Discussion**			
Key results	18	Summarise key results with reference to study objectives	10
Limitations	19	Discuss limitations of the study, taking into account sources of potential bias or imprecision. Discuss both direction and magnitude of any potential bias	14
Interpretation	20	Give a cautious overall interpretation of results considering objectives, limitations, multiplicity of analyses, results from similar studies, and other relevant evidence	10-14
Generalisability	21	Discuss the generalisability (external validity) of the study results	15
**Other information**			
Funding	22	Give the source of funding and the role of the funders for the present study and, if applicable, for the original study on which the present article is based	21

*Give information separately for exposed and unexposed groups.

Note: An Explanation and Elaboration article discusses each checklist item and gives methodological background and published examples of transparent reporting. The STROBE checklist is best used in conjunction with this article (freely available on the Web sites of PLoS Medicine at http://www.plosmedicine.org/, Annals of Internal Medicine at http://www.annals.org/, and Epidemiology at http://www.epidem.com/). Information on the STROBE Initiative is available at http://www.strobe-statement.org.

For peer review only

BMJ Open

The association between tuberculosis and pregnancy outcomes: A retrospective cohort study of women in Cape Town, South Africa.

Journal:	*BMJ Open*
Manuscript ID	bmjopen-2023-081209.R1
Article Type:	Original research
Date Submitted by the Author:	13-Dec-2023
Complete List of Authors:	Meehan, S; Stellenbosch University Faculty of Medicine and Health Sciences, Desmond Tutu TB Centre, Department of Paediatrics and Child Health<br>Hesseling, A C; Stellenbosch University Faculty of Medicine and Health Sciences, Desmond Tutu TB centre, Department of Paediatrics and Child Health<br>von Delft, Arne; University of Cape Town Department of Medicine, Centre for Infectious Disease Epidemiology and Research (CIDER), School of Public Health and Family Medicine; Western Cape Department of Health, Health Intelligence Directorate<br>Marx, Florian M.; Stellenbosch University Faculty of Medicine and Health Sciences, Desmond Tutu TB centre, Department of Paediatrics and Child Health; Heidelberg university hospital, Division of Infectious Disease and Tropical Medicine, Centre for Infectious Diseases<br>Hughes, Jennifer A.; Stellenbosch University, Desmond Tutu TB Centre, Department of Paediatrics and Child Health<br>Bock, Peter; Stellenbosch University Faculty of Medicine and Health Sciences, Desmond Tutu TB Centre, Department of Paediatrics and Child Health<br>Banke-Thomas, Aduragbemi; University of Greenwich; London School of Hygiene & Tropical Medicine, Faculty of Epidemiology and Population Health<br>Dunbar, R.; University of Stellenbosch, Desmond Tutu TB Centre, Department of Paediatrics and Child Health<br>Phelanyane, Florence; University of Cape Town Faculty of Health Sciences, Centre for Infectious Disease Epidemiology and Research (CIDER), School of Public Health and Family Medicine; Western Cape Department of Health, Health Intelligence Directorate, Department of Health and Wellness<br>Smith, Mariette; Western Cape Department of Health, Health Intelligence Directorate, Department of Health and Wellness ; University of Cape Town Faculty of Health Sciences, Centre for Infectious Disease Epidemiology and Research (CIDER), School of Public Health and Family Medicine<br>Osman, Muhammad; Stellenbosch University Faculty of Medicine and Health Sciences, Desmond Tutu TB Centre, Department of Paediatrics and Child Health; University of Greenwich, School of Human Sciences, Faculty of Education, Health and Human Sciences

**Primary Subject Heading**:	Public health
Secondary Subject Heading:	Infectious diseases, Health services research
Keywords:	Tuberculosis < INFECTIOUS DISEASES, Maternal medicine < OBSTETRICS, HIV & AIDS < INFECTIOUS DISEASES

SCHOLARONE™ Manuscripts

I, the Submitting Author has the right to grant and does grant on behalf of all authors of the Work (as defined in the below author licence), an exclusive licence and/or a non-exclusive licence for contributions from authors who are: i) UK Crown employees; ii) where BMJ has agreed a CC-BY licence shall apply, and/or iii) in accordance with the terms applicable for US Federal Government officers or employees acting as part of their official duties; on a worldwide, perpetual, irrevocable, royalty-free basis to BMJ Publishing Group Ltd ("BMJ") its licensees and where the relevant Journal is co-owned by BMJ to the co-owners of the Journal, to publish the Work in this journal and any other BMJ products and to exploit all rights, as set out in our licence.

The Submitting Author accepts and understands that any supply made under these terms is made by BMJ to the Submitting Author unless you are acting as an employee on behalf of your employer or a postgraduate student of an affiliated institution which is paying any applicable article publishing charge ("APC") for Open Access articles. Where the Submitting Author wishes to make the Work available on an Open Access basis (and intends to pay the relevant APC), the terms of reuse of such Open Access shall be governed by a Creative Commons licence – details of these licences and which Creative Commons licence will apply to this Work are set out in our licence referred to above.

Other than as permitted in any relevant BMJ Author's Self Archiving Policies, I confirm this Work has not been accepted for publication elsewhere, is not being considered for publication elsewhere and does not duplicate material already published. I confirm all authors consent to publication of this Work and authorise the granting of this licence.

For peer review only

The association between tuberculosis and pregnancy outcomes: A retrospective cohort study of women in Cape Town, South Africa.

Authors

Sue-Ann Meehan[1*], Anneke C. Hesseling[1], Arne von Delft[2,3], Florian M. Marx[1,4], Jennifer A. Hughes[1], Peter Bock[1], Aduragbemi Banke-Thomas[5,6], Rory Dunbar[1], Florence Phelanyane[2,3], Mariette Smith[2,3], Muhammad Osman[1,5]

*Corresponding author

Email address: sueannm@sun.ac.za (SM)

Affiliations:

[1]Desmond Tutu TB Centre, Department of Paediatrics and Child Health, Faculty of Medicine and Health Sciences, Stellenbosch University

[2]Centre for Infectious Disease Epidemiology and Research (CIDER), School of Public Health and Family Medicine, Faculty of Health Sciences, University of Cape Town

[3]Health Intelligence Directorate, Western Cape Government, Department of Health and Wellness

[4]Division of Infectious Disease and Tropical Medicine, Centre for Infectious Diseases, Heidelberg University Hospital, Heidelberg, Germany

[5]School of Human Sciences, Faculty of Education, Health and Human Sciences, University of Greenwich, London, United Kingdom

[6]Faculty of Epidemiology and Population Health, London School of Hygiene and Tropical Medicine, London, United Kingdom

Running Head: Pregnancy outcomes among women with TB

Key words: Maternal health, pregnancy outcomes, Tuberculosis, outcomes, linkage to care, HIV

Journal: BMJ Open

Word count: Abstract: 299/300 Main text: 3999/4000

For peer review only - http://bmjopen.bmj.com/site/about/guidelines.xhtml

Abstract

Background

Tuberculosis (TB) remains a leading cause of mortality among women of childbearing age and a significant contributor to maternal mortality. Pregnant women with tuberculosis are at high risk of adverse pregnancy outcomes. This study aimed to determine risk factors for an adverse pregnancy outcome among pregnant women diagnosed with tuberculosis,

Methods

Using tuberculosis programmatic data, this retrospective cohort analysis included all women who were routinely diagnosed with tuberculosis in the public sector between October 2018 and March 2020 in two health sub-districts of Cape Town, and who were documented to be pregnant during their tuberculosis episode. Adverse pregnancy outcome was defined as either a live birth of an infant weighing <2500g and/or with a gestation period <37 weeks or as stillbirth, miscarriage, termination of pregnancy, maternal or early neonatal death. Demographics, TB and pregnancy characteristics were described by HIV status. Logistic regression was used to determine risk factors for adverse pregnancy outcome.

Results

Of 248 pregnant women, half (52%) were living with human immunodeficiency virus (HIV); all were on antiretroviral therapy (ART) at the time of their tuberculosis diagnosis. Pregnancy outcomes were documented in 215 (87%) women, of whom 74 (34%) had an adverse pregnancy outcome. Being older (35-44 years vs 25-34 years (aOR: 3.99; 95% CI:1.37-11.57), living with HIV (aOR: 2.72; 95%CI: 0.99-4.63), having an unfavourable TB outcome (aOR 2.29; 95% CI: 1.03-5.08), and having presented to antenatal services ≤1 month prior to delivery (aOR 10.57: 95% CI:4.01-27.89) were associated with higher odds of an adverse pregnancy outcome.

Conclusions

Pregnancy outcomes among women with TB were poor, irrespective of HIV status. Pregnant women with TB are a complex population who need additional support prior to, during and after TB treatment to improve TB treatment and pregnancy outcomes. Pregnancy status should be considered for inclusion in TB registries.

For peer review only - http://bmjopen.bmj.com/site/about/guidelines.xhtml

Strengths and Limitations

- Using the Provincial Health Data Centre (PHDC), enabled the use of routine programmatic data from multiple integrated sources, that allowed us to evaluate pregnancy and TB outcomes with consideration of HIV.
- The PHDC enabled the use of an expanded definition of 'unfavourable TB treatment outcome', which more accurately reflects 'linkage to care' within the TB program for pregnant women.
- We could not determine a pregnancy outcome for 13% of our cohort, due to missing data, which is a limitation when using routine data.
- The data extract used for this analysis included all persons with TB, which meant that we were not able to compare pregnancy outcomes for women who did not have TB.

For peer review only - http://bmjopen.bmj.com/site/about/guidelines.xhtml

Background

Globally, 10.6 million people developed tuberculosis (TB) in 2021, of whom 3.4 million (32.1%) were women(1). TB incidence peaks in women during the reproductive years,(2,3) is a leading cause of mortality among women of childbearing age(4) and a significant contributor to maternal mortality(5). Pregnant women with TB are at high risk of adverse pregnancy outcomes including prematurity, low birthweight and stillbirth(6–8).

TB is common in settings with high HIV prevalence, especially in sub-Saharan Africa. People living with human immunodeficiency virus (HIV) have a 2.5 times increased risk of developing TB despite access to antiretroviral therapy (ART),(9) and TB remains the leading cause of death among people living with HIV(10). Pregnant women with TB and HIV are at increased risk of adverse pregnancy outcomes(11,12) and higher maternal and infant mortality(13–16). There are limited data on TB treatment outcomes amongst pregnant women since pregnancy status is not routinely captured or reported as a TB programme indicator globally and pregnancy testing results are not routinely captured for women with TB. While ART(17) and TB preventive treatment (TPT)(18) reduces the risk of TB disease, an estimated 150,600 pregnant and 49,000 postpartum women develop TB annually, with women in Africa accounting for 40% of this burden(19). There is a need to integrate routine pregnancy indicators in TB programmes to improve surveillance of pregnant women with TB.

In high TB burden settings, the World Health Organization (WHO) recommends screening pregnant women for TB at every contact with a healthcare worker(20). In South Africa, routine testing of all pregnant women for TB and providing TPT once TB disease is excluded is recommended(18) but the extent of implementation is unknown and most likely incomplete. There is a pressing need for improved data from routine and programmatic sources to identify programmatic gaps and inform evidence-based research interventions to improve health outcomes for pregnant women with TB and their infants. This study used routine data across three health programs (TB, maternal health, and HIV services) to determine risk factors associated with an adverse pregnancy outcome for women with TB.

For peer review only - http://bmjopen.bmj.com/site/about/guidelines.xhtml

Methods

Setting

In 2021, the estimated TB incidence in South Africa was 513 cases per 100,000 population(1) and HIV prevalence for adults (15+ years) was 17%(21). A third of women who received antenatal care in the public sector were living with HIV in 2019(22) and the HIV prevalence among persons with TB was 57% in 2021.(1) Maternal mortality was 139.3 deaths per 100,000 live births (2007-2015)(23). Although mortality among people with TB had declined between 2007 and 2016, approximately 12% of people with TB died in 2016.(24) TB was responsible for 11% of maternal deaths between 2014 and 2016 in South Africa(24). Maternal mortality for the Western Cape province ranged between 43.6 and 66.8 per 100 000 live births at healthcare facilities in 2017-2019(25).

We used routine health data from two large health sub districts (Khayelitsha and Tygerberg) in the Cape Metro, Western Cape Province, South Africa. The Cape Metro has a population of approximately 4.6 million people (two thirds of the provincial population); HIV/AIDS and TB were among the top six causes of death in 2015(26). The antenatal HIV prevalence was 22% in 2019(27). There were 26,000 newly diagnosed persons with TB and more than 2,000 TB deaths in 2022(28). Khayelitsha is a peri-urban low resourced health sub district, with a mix of formal and informal housing (29). It is serviced by a district hospital and 10 primary healthcare (PHC) facilities(26). Tygerberg is a large health subdistrict, (29) with mostly formal dwellings. It has a tertiary hospital, a district level hospital and 21 PHC facilities(26).

Study design

This was a retrospective cohort study based on programmatic data collected as part of a larger health system strengthening study (LINKEDin), which aimed to reduce initial loss to follow-up for people diagnosed with TB in three South African provinces(30).

Study population

We included data for all women who were routinely diagnosed with TB at either a hospital or PHC in the two health subdistricts between October 2018 and March 2020 and who were documented as having a pregnancy that overlapped with their TB episode (See Table 1 for definition of key terms), as recorded in routine health records.

For peer review only - http://bmjopen.bmj.com/site/about/guidelines.xhtml

Data collection

Data were extracted from the Western Cape Provincial Health Data Centre (PHDC). The PHDC is a comprehensive linked health information exchange in the province and uses unique identifiers to integrate all electronic health data from routine health information systems in the public sector (laboratory, pharmacy, administrative and other clinical data) into single patient level records to support improved patient care(31). PHDC outputs include line lists (cascades) of patients with specific conditions e.g. TB, HIV, pregnancy(31). These are updated daily and can be used to track patients at each healthcare visit and identify those requiring follow-up, including those newly diagnosed with TB who have not yet been entered into a TB treatment register and initiated on TB treatment.

We accessed the de-identified TB cascade from the PHDC and filtered for pregnancy status, to identify all women with TB who were pregnant during the study period in the two sub-districts. We used the unique identifier for these individuals to access data contained in the HIV and pregnancy cascades. Cascades are individual patient level views of specific health conditions which include key dates, evidence, outcomes, and co-morbidities relevant for patient management. We merged the three cascades and checked our dataset for consistency, confirming that all women included in our cohort had a pregnancy that overlapped with their TB episode (Table 1).

We extracted demographic (age) and clinical variables for TB, HIV, and pregnancy (e.g., *Mycobacterium tuberculosis* drug susceptibility status, HIV status, CD4 count, gravidity, infant birthweight etc) and key dates (date of TB diagnosis, date first known to the public antenatal services, date of delivery etc). To determine TB treatment outcomes, we reviewed the evidence recorded in the PHDC relating to evidence of linkage to a TB treatment facility and if linked, we extracted their TB treatment initiation and outcome data (cured, successfully completed, treatment failure, loss to follow up, died, not evaluated). We allocated a TB treatment outcome (favourable or unfavourable) as per our definition (Table 1). We used an expanded definition of unfavourable TB outcome to include newly diagnosed persons with TB who did not link to care and initiate treatment at a TB treatment facility. To determine a pregnancy outcome, we reviewed live birth,

For peer review only - http://bmjopen.bmj.com/site/about/guidelines.xhtml

infant birthweight, and gestational period and allocated a pregnancy outcome (Good or adverse), as per definitions in Table 1. Women who had one or more of these variables missing, had a pregnancy outcome of unknown asssigned. Pregnancy outcomes could occur either during or after the end of the TB episode (Figure 1).

Power calculation

Based on a sample of 250 women including 170 with favourable TB outcomes and 80 with unfavourable TB outcomes, we estimated that we would have 89% power with 95% significance to detect a difference of 20 percentage points in adverse pregnancy outcomes by TB outcomes.

Patient and Public involvement

As this study used routine data at the programme level, patients were not directly involved in any aspect of the study. We did engage our institutional community advisory board (CAB), on the design and implementation strategies of the broader overall study (LINKEDin), which primarily involved the evaluation of two interventions to reduce initial loss to follow up among TB patients in South Africa. In this sub analysis we used standardised TB and pregnancy outcomes. We presented our findings to the CAB prior to writing this manuscript and then again more recently to get their input on a proposed future study to address poor TB and pregnancy outcomes among this complex population.

Statistical analysis

Descriptive statistics were used to describe baseline characteristics, the timing of pregnancy and delivery compared to the TB episode, stratified by HIV status. A logistic regression model was used to determine risk factors for adverse pregnancy outcomes including TB treatment outcomes and other predictors. Predictors were added incrementally, observing the change in significance of the likelihood ratio test of each model, to produce a final adjusted model. The relationship between predictors was considered and we avoided co-linearity in the final model. SAS software (version 9.4; SAS Institute, Inc, Cary, NC) was used for analysis.

Ethical considerations

For peer review only - http://bmjopen.bmj.com/site/about/guidelines.xhtml

The Health Research Ethics Committee of Stellenbosch University (N18/07/069) approved the study, which was conducted according to the guiding principles within the Declaration of Helsinki. Approvals were received from the Western Cape Department of Health and the City of Cape Town Health Directorate. We received a waiver of individual consent for this routinely collected health data.

Results

Overall

A total of 260 pregnant women were diagnosed with TB between October 2018 and March 2020, of whom 67 had a pregnancy recorded prior to their TB diagnosis. We excluded 12 women because their pregnancy outcome was also recorded prior to their TB diagnosis. There were 111 women who had their pregnancy outcome documented before their TB outcome (i.e. who delivered while still on TB treatment), and 137 women who had their pregnancy outcome documented after their TB outcome (i.e. who delivered after their TB treatment). See Figure 1.

Among the 248 women included in analysis, the median age was 27.7 years (IQR: 22.7-32.3), range 14-43 years. The majority of women (191/248; 77%) had bacteriologically confirmed TB, 170/248 (69%) were treated for drug-susceptible TB and 10/248 (4%) were treated for drug-resistant TB. All had an HIV status recorded, half (130/248; 52%) were living with HIV. Women living with HIV (WLWH) were slightly older (29.1 years; IQR 25.7-33.4) compared to HIV negative women (25.4 years; IQR 20.6-30.1). Of WLWH, the median CD4 count at TB diagnosis was 236 cells/µL(IQR:127-423) and all were on antiretroviral therapy (ART) at the time of their TB diagnosis, with 56/130 (43%) having initiated ART during their pregnancy. Supplementary Table S1 provides characteristics of pregnant women with HIV by pregnancy status at the time ART was initiated. Overall, 9/248 of women (4%) died, of whom 6/9 (66%) were living with HIV. Most women (6/9) died >60 days after their TB diagnosis had been made (Table 2).

For half of the women (121/248; 49%), this was their first pregnancy. Most (193/248; 78%) had their pregnancy recorded at or after the time their TB diagnosis was made, with 64% (124/193) delivering after their TB outcome was documented (Figure 1). A third (83/248; 33%) were documented to have attended antenatal services late, presenting for

For peer review only - http://bmjopen.bmj.com/site/about/guidelines.xhtml

the first visit within 30 days of their delivery, with most of these women (67/83; 81%) presenting to services within two days of delivering their infant. Overall, the median gestation time was 40 weeks (IQR 35-40 weeks). Amongst infants overall, the median birth weight was 2875 grams (IQR:2445-3270 grams) (Table 2).

TB treatment outcomes

Using the more comprehensive definition including initial loss to follow-up, a third (79/248; 32%) of pregnant women had an unfavourable TB treatment outcome; 44/130 (33.8%) among those living with HIV and 35/118 (29.7%) who were HIV negative. Of those with an unfavourable TB treatment outcome, 34% (27/79) were never linked to TB care and were not recorded to have started TB treatment in a TB treatment register. Of those not linked to care, a quarter (7/27; 26%) died (3 HIV negative and 4 HIV positive) (Figure 2).

Pregnancy outcomes

Pregnancy outcomes were documented in 215/248 (86.7%) women. Overall, 74/215 (34.4%) women had an adverse pregnancy outcome; 40/106 (37.7%) among WLWH and 34/109 (31.2%) among HIV negative women. Overall, adverse pregnancy outcomes were higher among women who had unfavourable TB treatment outcomes (32/79; 40.5%) vs. favourable TB treatment outcomes (42/169; 24.9%), irrespective of HIV status (Figure 2).

Risk factors associated with an adverse pregnancy outcome

Pregnant women with TB were at increased odds of having an adverse pregnancy outcome if they were older [adjusted odds ratio (aOR) 3.99: 95% confidence interval (CI):1.37-11.57], living with HIV (aOR 2.72: 95%CI 1.2-6.18), had an unfavourable TB treatment outcome (aOR 2.29: 95%CI: 1.03-5.08) and if they presented late to antenatal services (≤30 days prior to their delivery), compared to attending antenatal services for at least 121 days (aOR 10.57: 95%CI:4.01-27.89). Women were less likely to have an adverse pregnancy outcome with increasing gravidity (aOR 0.55: 95%CI:0.37-0.81) (Table 3).

Discussion

For peer review only

For peer review only - http://bmjopen.bmj.com/site/about/guidelines.xhtml

This study included a large cohort of women diagnosed with TB in routine services with a pregnancy that overlapped their TB episode. Pregnancy outcomes were poor; being older, living with HIV, having an unfavourable TB treatment outcome and presenting late to antenatal services were all risk factors associated with an adverse pregnancy outcome. Half of these women were living with HIV, higher than the estimated antenatal HIV prevalence rate of 22% in the setting(27). All HIV-positive women were on ART; more than half (57%) were already on ART prior to their pregnancy, with the balance initiating ART during their pregnancy. Overall, these findings indicate good HIV screening and ART initiation services generally and specifically among pregnant women attending public antenatal services. As comparison, approximately 54% of all people living with HIV were on ART in 2019 in Cape Town (32).

Overall, 34% of women with TB had an adverse pregnancy outcome. A systematic review of 69 studies (published 2009-2021), the majority of which were from low-and-middle-income countries, reported poor pregnancy outcomes for women with TB, with most studies reporting low birthweight and/or preterm labour and/or spontaneous abortions(33). A South African study reported 48% of women routinely treated for MDR/RR-TB during 2013 to 2017, had a poor pregnancy outcome,(34) higher than our study, where the majority had drug-sensitive TB. Other South African studies with cohorts between 2011(15) and 2014(14) showed that of infants born to HIV-positive mothers with TB, between 20% and 70% had a gestation period <37 weeks and between 21% and 59% had a low birth weight. Since these previous studies, there have been many substantial advances and positive policy changes in our setting including universal HIV testing, universal ART rollout(35) and pregnant women being eligible for TB preventive therapy irrespective of HIV status(18). Yet our findings show similar high rates of poor pregnancy outcomes in women, irrespective of HIV status. This is highly concerning. Our data highlights the need for additional support and interventions for pregnant women with TB, including strategies to improve the integration of TB and maternal services to improve TB and pregnancy outcomes for these women. Additional work is needed to understand the care pathway for these women, barriers and enablers to care and determine gaps between policy and implementation. We also note that comparison across studies is challenging as the definition of an adverse pregnancy outcome varied by study. A

For peer review only - http://bmjopen.bmj.com/site/about/guidelines.xhtml

standardized definition including all variables that affect both maternal and infant outcomes is needed.

Having a poor TB treatment outcome was associated with having a poor pregnancy outcome, which is biologically plausible. We are not aware of other studies that have reported on poor TB treatment outcomes as a risk factor for adverse pregnancy outcomes. A study among pregnant women with MDR/RR-TB found that 40% had an unfavourable TB treatment outcome, as per traditional WHO definitions(7). This study found a third of women had an unfavourable TB outcome, but used a broader definition to include those lost prior to registration/treatment initiation(36). This study identified a high proportion of women who did not link to TB care; 43% among HIV-negative women and 27% among HIV-positive women. For persons with TB in South Africa, approximately 12% among drug-sensitive(37) and 37% among drug-resistant(38) persons with TB are never started on treatment. Considering that the majority of our cohort had drug-sensitive TB, loss prior to registration/treatment initiation among our cohort was higher than what is reported for persons with TB overall. As these women are not recorded in TB treatment registers, their data is not included in routine recording and reporting, further excerbating the gaps in surveillance of TB in women of childbearing age.

Pregnant women diagnosed with TB are a complex population and require more intensive follow up to ensure better TB and pregnancy outcomes, and to reduce mortality. We recommend improved one-on-one communication by healthcare workers when referring these women to care(39). Skilled personnel should ensure that women understand their disease and the importance of linking to ongoing care and treatment. More broadly, health services should update contact details of persons with TB at each health visit, so that they can be easily traced if they do not link to TB care. This is especially important for pregnant women with TB, who may have additional barriers to accessing care. Routine antenatal and postnatal services should serve as an important trigger to link women to TB treatment following screening and testing. Being lost to the health system after a TB diagnosis may be the underlining reason for having an adverse pregnancy outcome. The association between TB and pregnancy outcomes should be further explored to better understand how these are associated.

For peer review only - http://bmjopen.bmj.com/site/about/guidelines.xhtml

Most women had their pregnancy recorded only after they were diagnosed with TB. This is an interesting finding, as typically women are screened for TB during antenatal visits. This finding may be highlighting a need for improved sexual and reproductive health counselling with women who are accessing TB care in this setting. We are also mindful of the limitations of using routine data and that the capturing of the first date known to the antenatal services may not always be accurate. This finding should be interpreted with caution. Offering effective contraception to women at TB treatment initiation(33,40) and screening for pregnancy at each TB-related visit, with integration of reproductive health services as part of TB care is critically important(41). In addition to counselling on contraceptive services, women should be made aware of their increased risk of poorer pregnancy outcomes and the need for additional care if they conceive during their TB episode. If adequate counselling and assessment of family planning at the start of TB treatment is being implemented as per local guidelines, and women continue to fall pregnant during their TB episode, then there could be other factors associated with this. Socio-behavioural research may elicit such factors and provide more person-centered understanding that could guide better implementation of exitsing or more patient-centered policies .

Living with HIV was found to be a risk factor for having an adverse pregnancy outcome, similar to previous studies. In our study, women had similar poor pregnancy outcomes irrespective of whether ART was started prior to or during pregnancy. A systematic review, including 73 cohort studies found that ART reduced the risk of adverse perinatal outcomes in pregnant women living with HIV, but the risk remained higher than in HIV-negative women(42). South African studies have shown that although increased ART coverage improved birth outcomes, HIV-positive women were significantly more likely to have a stillbirth or premature infant compared to HIV-negative women(43) and were at increased risk of having a ‘small for gestational age’ infant, regardless of when they had initiated ART(44). While pregnancy remains an excellent opportunity to start ART, the WHO recommends the immediate initiation of lifelong ART for all people living with HIV(45). Despite a universal test and treat policy for HIV, the findings from our study show that many women start ART only at antenatal presentation. A significant improvement in community-based HIV testing and treatment to support earlier ART initiation is essential.

For peer review only - http://bmjopen.bmj.com/site/about/guidelines.xhtml

ART initiation prior to pregnancy may reduce adverse pregnancy outcomes for these women and also TB treatment outcomes.

Our findings show that a third of women (25% of women living with HIV and 42% of HIV-negative women) were only documented to have sought care at public antenatal services during the month prior to delivery, highlighting the challenge of 'late booking'. All antenatal, TB and HIV services are free of direct charge in South Africa in the public sector. This proportion is higher than in the 2019 South African Antenatal survey, where only a small proportion of women (5.6%) attended their first antenatal visit in the last trimester of their pregnancy(22). In the Cape Town Metro, 75% of women attend antenatal services before 20 weeks(27). South Africa guidelines indicate that booking should be as early as possible (46) to ensure optimal antenatal care. Clearly, pregnant women with TB are an especially high-risk group overall. Those who book late should be tested for TB as a priority and offered TPT. Tracking pregnancy using 'action lists' may offer the opportunity for personnel in the TB programme to create awareness around the importance of adequate antenatal visits and urge these women to access maternal services earlier in their pregnancy.

We found that gravidity had a protective effect. This could be an example of the "healthy mother effect," where women who have had successful pregnancies in the past are more likely to have successful pregnancies in the future(47). This finding should be interpreted with caution.

Our study addresses a key yet neglected aspect of TB and maternal child health, using routine health data from multiple integrated sources that allowed us to evaluate pregnancy and TB treatment outcomes with consideration of HIV. We demonstrate poor pregnancy outcomes in pregnant women with TB, half of whom were also living with HIV. Using the PHDC strengthened our ability to determine more inclusive definitions of favourable and unfavourable TB treatment outcomes and enabled us to include all pregnant women diagnosed with TB, so we could determine both pre- and post-loss to follow up during TB care. Using our expanded definition of an 'unfavourable' TB treatment outcome, this study was able to reflect more accurately 'linkage to care' within the TB program for pregnant women. From a program perspective, having a maternal cascade is

For peer review only - http://bmjopen.bmj.com/site/about/guidelines.xhtml

a benefit for the monitoring of the maternal and infant health program in services. Health services should allow for integrated tools to provide optimal care for patients across multiple programs: maternal, TB and HIV programs.

We could not determine a pregnancy outcome for 13% of our cohort, due to missing data. This may be due to women delivering outside the province, data linkage limitations between mother and infant, or incomplete data capture, potentially resulting in a biased outcome and over-reporting of adverse pregnancy outcomes. Missing data is a limitation when using routine health data sources. A second limitation is that we were unable to compare pregnancy outcomes for our cohort to women who did not have TB during their pregnancy. This analysis was nested within a larger study and our data extract was restricted to persons with TB. Future work should compare pregnancy outcomes for women by TB status and TB outcomes by pregnancy status. A third limitation is that while the multivariable regression model was able to control for selected factors, residual confounding could have occurred due to other maternal factors (gestational diabetes, hypertension, anaemia) or socio-economic factors (poverty, substance use, educational status). These data were not available for analysis and should be included in future work. A further limitation is the inability to explain the time between presenting with TB and the timing of pregnancy.

Conclusions

Pregnancy outcomes among women with TB were poor, irrespective of HIV status. This is a major concern. TB care needs to be strengthened for pregnant women and health services need to think through how best to optimise maternal services within the TB program. We used routine data to track women across the TB, HIV and maternal healthcare cascades, allowing for more inclusive definitions and analysis of linkage to care and TB treatment and pregnancy outcomes. Our study highlights the high proportion of women diagnosed with TB who never started TB treatment and the large proportion of women who only sought antenatal care services during the month prior to delivery. Pregnant women with TB are a complex population who need additional support prior to, during and after TB treatment to improve pregnancy outcomes, irrespective of HIV status. Going forward, recording of pregnancy status should be a key indicator piloted as part of

For peer review only - http://bmjopen.bmj.com/site/about/guidelines.xhtml

TB treatment registries, as this would result in better quality data and offer opportunites for earlier intervention and support of pregnant and postpartum women with TB.

For peer review only - http://bmjopen.bmj.com/site/about/guidelines.xhtml

Figure legends

Figure 1: Timing of pregnancy in relation to the tuberculosis episode

Figure 2: Tuberculosis outcomes and pregnancy outcomes by HIV status (n=248)

Acknowledgements

We wish to acknowledge our implementing partners; the University of Cape Town and the Centre for Infectious Disease Epidemiology and Research (CIDER) in the Western Cape Province. We further acknowledge the staff at the Western Cape Provincial Health Data Centre (PHDC) for their invaluable assistance. We highly appreciate input from the health staff at the provincial, district and sub-district health offices and those at the facilities in which the study was implemented.

Contributorship statement

SM and MO planned and designed the study, were involved in data collection, extraction, and validation. MO produced the analysis. SM and MO interpreted the data.

SM wrote the first draft and finalised the submission based on author and peer reviewer input.

AvD, RD, FP and MS were involved in data collection, extraction, and validation, interpretation of results, reviewed the manuscript and provided critical input.

ACH, FM, JH, PB, AB-T were involved in data interpretation, reviewed the manuscript and provided critical input.

All authors have reviewed the final version of the manuscript and approve of its content and submission for publication.

Conflict of Interest

The authors have no conflict of interest to declare.

Funding

This research study and publication was supported by the Bill and Melinda Gates Foundation (BMGF), Investment INV- 007130. The contents are the responsibility of the authors and do not necessarily reflect the views of the BMGF. The funders had no role in study design, data collection and analysis, decision to publish, or preparation of the manuscript.

For peer review only - http://bmjopen.bmj.com/site/about/guidelines.xhtml

ACH is funded by the South African National Research Foundation SaRchi Chair in Paediatric Tuberculosis.

Data sharing statement

The de-identified dataset is available from the first author, on request. This analysis was nested within a broader study and the dataset is still being analysed within the broader study and will only be made public after the primary paper has been published.

Ethical statement

Stellenbosch University Health Reseach Ethics Committee (N18/07/069). The study included routine programmatic data and so did not include human participants directly.

For peer review only

For peer review only - http://bmjopen.bmj.com/site/about/guidelines.xhtml

References

1. World Heath Organization. Global tuberculosis report 2022 [Internet]. Geneva; Available from: https://www.who.int/teams/global-tuberculosis-programme/tb-reports/global-tuberculosis-report-2022
2. Deluca A, Chaisson R, Martinson N. Intensified case finding for tuberculosis in prevention of mother-to-child transmission programs: A simple and potentially vital addition for maternal and child health. J Acquir Immune Defic Syndr. 2009;50(2):196–9.
3. Gupta A, Nayak U, Ram M, Bhosale R, Patil S, Basavraj A, et al. Postpartum tuberculosis incidence and mortality among HIV-infected women and their infants in Pune, India, 2002-2005. Clin Infect Dis. 2007;45(2):241–9.
4. Zumla A, Bates M, Mwaba P. The neglected global burden of tuberculosis in pregnancy. Lancet Glob Heal [Internet]. 2014;2(12):e675–6. Available from: http://dx.doi.org/10.1016/S2214-109X(14)70338-9
5. Loto OM, Awowole I. Tuberculosis in pregnancy: A review. J Pregnancy. 2012;2012(379271).
6. Alene KA, Murray MB, van de Water BJ, Becerra MC, Atalell KA, Nicol MP, et al. Treatment Outcomes Among Pregnant Patients With Multidrug-Resistant Tuberculosis. JAMA Netw Open. 2022;5(6):e2216527.
7. Mokhele I, Jinga N, Berhanu R, Dlamini T, Long L, Evans D. Treatment and pregnancy outcomes of pregnant women exposed to second-line anti-tuberculosis drugs in South Africa. BMC Pregnancy Childbirth [Internet]. 2021;21(1):1–11. Available from: https://doi.org/10.1186/s12884-021-03956-6
8. Jana N, Barik S, Arora N, Singh AK. Tuberculosis in pregnancy: The challenges for South Asian countries. J Obstet Gynaecol Res. 2012;38(9):1125–36.
9. Yaghoubi A, Salehabadi S, Abdeahad H, Hasanian SM, Avan A, Yousefi M, et al. Tuberculosis, human immunodeficiency viruses and TB/HIV co-infection in pregnant women: A meta-analysis. Clin Epidemiol Glob Heal. 2020;8(4):1312–20.
10. Zumla A, Malon P, Henderson J, Grange JM. Impact of HIV infection on tuberculosis. Postgr Med J. 2000;76:259–68.
11. Rollins NC, Coovadia HM, Bland RM, Coutsoudis A, Bennish ML, Patel D, et al. Pregnancy outcomes in HIV-infected and uninfected women in rural and urban South Africa. J Acquir Immune Defic Syndr. 2007;44(3):321–8.

For peer review only - http://bmjopen.bmj.com/site/about/guidelines.xhtml

12. Arab K, Spence AR, Czuzoj-Shulman N, Abenhaim HA. Pregnancy outcomes in HIV-positive women: a retrospective cohort study. Arch Gynecol Obstet. 2017;295(3):599–606.

13. Calvert C, Ronsmans C. The contribution of HIV to pregnancy-related mortality: A systematic review and meta-analysis. Aids. 2013;27(10):1631–9.

14. Salazar-Austin N, Hoffmann J, Cohn S, Mashabela F, Waja Z, Lala S, et al. Poor Obstetric and Infant Outcomes in Human Immunodeficiency Virus-Infected Pregnant Women with Tuberculosis in South Africa: The Tshepiso Study. Clin Infect Dis. 2018;66(6):921–9.

15. Bekker A, Schaaf HS, Draper HR, Kriel M, Hesseling AC. Tuberculosis disease during pregnancy and treatment outcomes in HIV-infected and uninfected women at a referral Hospital in Cape Town. PLoS One. 2016;11(11):1–14.

16. Mnyani CN, McIntyre JA. Tuberculosis in pregnancy. BJOG An Int J Obstet Gynaecol. 2011;118(2):226–31.

17. Lawn SD, Harries AD, Williams BG, Chaisson RE, Losina E, Cock KM De, et al. Antiretroviral therapy and the control of HIV-associated tuberculosis. Will ART do it? Int J Tuberc Lung Dis. 2011;15(5):571–81.

18. South African National Department of Health. National Guidelines on the Treatment of Tuberculosis Infection [Internet]. 2023. Available from: https://www.knowledgehub.org.za/system/files/elibdownloads/2023-03/Health_Latent TB Infection_2023_web.pdf

19. Mafirakureva N. Revisiting the burden of TB in pregnant and post-partum women. In: International Journal of Tuberculosis and Lung Disease Abstract book 50th World Conference Hyderabad, India [Internet]. 2019. Available from: https://theunion.org/our-work/conferences/history-of-the-union-world-conference-on-lung-health/conference-abstract-books

20. World Health Oghanization. WHO consolidated guidelines on tuberculosis Module 2: Screening Systemic screening for tuberculosis disease [Internet]. World Health Organization. 2020. Available from: https://apps.who.int/iris/handle/10665/340255

21. UNAIDS:https://aidsinfo.unaids.org/. Aids Information [Internet]. 2020. [cited 2020 May 11]. Available from: https://aidsinfo.unaids.org

22. Woldesenbet, S.A., Lombard, C., Manda, S., Kufa, T., Ayalew, K., Cheyip M., and Puren A. The 2019 National Antenatal Sentinel HIV Survey, South Africa, National

For peer review only - http://bmjopen.bmj.com/site/about/guidelines.xhtml

Department of Health [Internet]. 2021. Available from: https://www.nicd.ac.za/wp-content/uploads/2021/11/Antenatal-survey-2019-report_FINAL_27April21.pdf

23. Bomela NJ. Maternal mortality by socio-demographic characteristics and cause of death in South Africa: 2007-2015. BMC Public Health. 2020;20(1):1–20.

24. Loveday M, Mzobe YN, Pillay Y, Barron P. Figures of the dead: A decade of tuberculosis mortality registrations in South Africa. South African Med J. 2019;109(10):728–32.

25. Kalk E, Heekes A, Slogrove AL, Phelanyane F, Davies MA, Myer L, et al. Cohort profile: the Western Cape Pregnancy Exposure Registry (WCPER). BMJ Open. 2022;12(6).

26. Western Cape Government: Health. Cape Metro Health District Plan (2021/22 to 2023/24). 2021.

27. Woldesenbet, S.A., Lombard, C., Manda, S., Kufa, T., Ayalew, K., Cheyip M., and Puren A. The 2019 National Antenatal Sentinel HIV Survey, South Africa, National Department of Health. 2021.

28. Western Cape Government Department of Health. Western Cape Provincial Tuberculosis (TB) Dashboard [Internet]. 2022 [cited 2022 Oct 15]. Available from: https://www.westerncape.gov.za/site-page/provincial-tb-dashboard

29. Western Cape Government Health Department. Circular H 102/2020 Covid-19 Population Data. Chief Dir Strateg Heal Support [Internet]. 2020;1–123. Available from: https://www.westerncape.gov.za/assets/departments/health/h_102_2020_covid-19_population_data.pdf

30. Osman M, Meehan S-A, Delft A von, Boulle A, Dunbar R, Smith M, et al. Reducing initial loss to follow-up among people with TB in Cape Town, South Africa. In: 50th World Conference on Lung health International Union Against Tuberculosis and Lung Disease (The Union) Abstract Book [Internet]. 2021. Available from: https://repositorio.conacyt.gov.py/handle/20.500.14066/3521

31. Boulle A, Heekes A, Tiffin N, Smith M, Mutemaringa T, Zinyakatira, N, Phelanyane F, et al. Data Centre Profile: The Provincial Health Data Centre of the Western Cape. Int J Popul Data Sci. 2019;0(October).

32. Western Cape Government Department of Health. Cape Metro District Health Plan 2018/19 -2020/21 [Internet]. 2018. Available from: http://www.health.gov.za/DHP/docs/DHP2018-

For peer review only - http://bmjopen.bmj.com/site/about/guidelines.xhtml

21/Western_Cape/Metro_District_Health_Plan.pdf

33. Nguyen Y, McNabb KC, Farley JE, Warren N. Examining family planning and adverse pregnancy outcomes for women with active tuberculosis disease: a systematic review. BMJ Open. 2022;12(3):e054833.

34. Loveday M, Hughes J, Sunkari B, Master I, Hlangu S, Reddy T, et al. Maternal and Infant Outcomes among Pregnant Women Treated for Multidrug/Rifampicin-Resistant Tuberculosis in South Africa. Clin Infect Dis. 2021;72(7):1158–68.

35. South African National Department of Health. Circular: Implementation of the universal test and treat strategy for HIV positive patients and differentiated care for stable patients [Internet]. 2016. Available from: https://sahivsoc.org/Files/22 8 16 Circular UTT Decongestion CCMT Directorate.pdf

36. Linh NN, Viney K, Gegia M, Falzon D, Glaziou P, Floyd K, et al. World Health Organization treatment outcome definitions for tuberculosis: 2021 update. Eur Respir J [Internet]. 2021;58(2). Available from: http://dx.doi.org/10.1183/13993003.00804-2021

37. Naidoo P, Theron G, Rangaka MX, Chihota VN, Vaughan L, Brey ZO, et al. The South African Tuberculosis Care Cascade: Estimated Losses and Methodological Challenges. J Infect Dis. 2017;216(Suppl 7):S702–13.

38. Cox H, Dickson-Hall L, Ndjeka N, van't Hoog A, Grant A, Cobelens F, et al. Delays and loss to follow-up before treatment of drug-resistant tuberculosis following implementation of Xpert MTB/RIF in South Africa: A retrospective cohort study. PLoS Med. 2017;14(2):1–19.

39. Viljoen L, Hendricks P, Hoddinott G, Vanqa N, Osman M, Hesseling AC, et al. Early interactions with newly diagnosed TB patients in hospital can support linkage to care. Public Heal Action [Internet]. 2022;12(3). Available from: http://dx.doi.org/10.5588/pha.22.0012%0A

40. Schnippel K, Ndjeka N, Conradie F, Berhanu R, Claasen Z, Banoo S, et al. A call to action: Addressing the reproductive health needs of women with drug-resistant tuberculosis. South African Med J. 2016;106(4):333–4.

41. Maugans C, Loveday M, Hlangu S, Waitt C, Schalkwyk M Van, Water B Van De, et al. Best practices for the care of pregnant people living with TB. Int J Lung Dis. 2023;27(January):357–66.

42. Portwood C, Sexton H, Kumarendran M, Brandon Z, Kirtley S, Hemelaar J. Adverse

For peer review only - http://bmjopen.bmj.com/site/about/guidelines.xhtml

perinatal outcomes associated with antiretroviral therapy in women living with HIV: A systematic review and meta-analysis. Front Med. 2023;9(February).

43. Cornish EF, Hudson J, Sayers R, Loveday M. Improving access to contraception through integration of family planning services into a multidrug-resistant tuberculosis treatment programme. BMJ Sex Reprod Heal. 2020;46(2):152–5.

44. Malaba TR, Mukonda E, Matjila M, Madlala HP, Myer L, Newell ML. Pregnancy outcomes in women living with HIV and HIV-negative women in South Africa: Cohort analysis based on bias-corrected gestational age. Paediatr Perinat Epidemiol. 2022;36(4):525–35.

45. World Heath Organization. Consolidated Guidelines on the use of Antiretroviral drugs for treating and preventing HIV infection. Recommendations for a public health approach. 2nd Edition. 2016.

46. South African National Department of Health. Guidelines for maternity care in South Africa. A manual for clinics, community health centres and district hospitals. Fourth Edition. [Internet]. 2016. Available from: https://knowledgehub.health.gov.za/elibrary/guidelines-maternity-care-south-africa-2016

47. Whitley E, Doyle P, Roman E, De Stavola B. The effect of reproductive history on future pregnancy outcomes. Hum Reprod. 1999;14(11):2863–7.

For peer review only

Table 1. Key variable definitions for TB, HIV, and pregnancy outcomes

**Definition of terminology**	
**TB episode**	Period from the date of TB diagnosis to the recorded date of TB outcome or death.
**TB diagnosis**	Includes a bacteriological (evidence of a positive TB laboratory test) or clinical diagnosis of TB (including evidence of TB drugs administered as part of a therapeutic regimen, or primary ICD10 codes and inclusion in a primary care TB register).
**Pregnancy episode**	The period from the first electronically captured evidence of a pregnancy documented in antenatal health services to the date of delivery or of any other pregnancy outcome.
**Overlapping pregnancy**	Date of the first evidence of the pregnancy was ≤9 months prior to the date of the TB diagnosis or ≤18 months after the date of the TB diagnosis
**TB treatment outcomes**	
Favourable TB outcome	Recorded in the routine health service data as treatment success (bacteriologically cured or successfully completed TB treatment).
Unfavourable TB outcome (expanded TB treatment outcome definition)	Recorded in the routine health service data as lost to follow up, died during treatment, treatment failed or not evaluated (WHO TB outcomes) OR loss to follow up prior to linkage to TB care (including died prior to linkage, i.e. "initial loss to follow-up").
**Linkage to TB care**	Evidence of accessing a TB treatment facility for TB treatment (TB hospital or a PHC facility offering TB treatment) after the diagnosis of TB.
**Pregnancy outcomes**	
Good pregnancy outcome	A pregnancy that resulted in the live birth of an infant weighing ≥2500g and with a gestation period ≥ 37 weeks.

For peer review only

Adverse pregnancy outcome	A pregnancy that resulted in either a live birth of an infant weighing <2500g and/or with a gestation period < 37 weeks or where there was a still birth, miscarriage, termination, the mother dying prior to delivery or early neonatal death.

For peer review only

Table 2. Demographic and clinical characteristics among pregnant women with tuberculosis, by HIV status (n=248).

	Total N (%)	HIV status n (%)	
	(N=248)	HIV+ (n=130)	HIV- (n=118)
**Age in years, median (IQR)**	27.7 (22.7-32.3)	29.1 (25.7-33.4)	25.4 (20.6-30.1)
14-17 years	10 (4.1)	3 (2.3)	7 (5.7)
18-24 years	75 (30.2)	25 (19.2)	50 (40.7)
25-34 years	129 (52.0)	82 (63.1)	47 (38.2)
35-44 years	34 (13.7)	20 (15.4)	14 (11.4)
**Pregnancy recorded in relation to TB diagnosis**			
Pregnant before TB diagnosed	55 (22.2)	33 (25.4)	22 (18.6)
Pregnant after TB diagnosed	193 (77.8)	97 (74.6)	96 (81.4)
**TB diagnosis (%)**			
Bacteriologically confirmed	191 (77.0)	93 (71.5)	98 (83.1)
Clinically diagnosed	57 (23.0)	37 (28.5)	20 (16.9)
**Place of TB diagnosis (%)**			
Hospital	78 (31.5)	46 (35.4)	32 (27.1)
PHC facility	170 (68.5)	84 (64.6)	86 (72.9)
**TB Treatment Category**			
New	162 (65.3)	85 (65.4)	77 (65.3)
Retreatment	66 (26.6)	37 (28.5)	29 (24.6)
Unknown	20 (8.1)	8 (6.2)	12 (10.2)
**Site of disease**			
Extra-pulmonary TB	44 (17.7)	32 (24.6)	12 (10.2)
Pulmonary TB	196 (79.0)	92 (70.8)	104 (88.1)
Not specified	8 (3.2)	6 (4.6)	2 (1.7)
**Drug susceptibility status**			
DR TB	10 (4.0)	5 (3.8)	5 (4.2)
DS TB	170 (68.5)	81 (62.3)	89 (75.4)
DST not recorded	68 (27.4)	44 (33.8)	24 (20.3)
**TB outcomes (%)**			
Favourable	169 (68.1)	86 (66.2)	83 (70.3)
Unfavourable	79 (31.9)	44 (33.8)	35 (29.7)
**Linked to TB Care**			
Linkage ≤30 days	202(81.5)	107 (82.3)	95 (80.5)
Linkage >30days	19 (7.7)	11 (8.5)	8 (6.8)
Unlinked	27 (10.9)	12 (9.2)	15 (12.7)
**Died**			
Yes	9 (3.6)	6 (4.6)	3 (2.5)
No	239 (96.4)	124 (95.4)	115 (97.5)
**Time to maternal death (from date of TB diagnosis)**			
0-30 days	3 (33.3)	1 (16.7)	2 (66.7)
30-59 days	0 (0)	0 (0)	0 (0)
61-90 days	2 (22.2)	2 (33.3)	0 (0)
≥90 days	4 (44.4)	3 (50.0)	1 (33.3)

For peer review only - http://bmjopen.bmj.com/site/about/guidelines.xhtml

**Parity**			
0	121 (48.8)	55 (42.3)	66 (55.9)
1	71 (28.6)	47 (36.2)	24 (20.3)
2	35 (14.1)	19 (14.63.8)	16 (13.6)
>2	21 (8.5)	9 (6.9)	12 (10.2)
**Gravidity**			
1	116 (46.8)	52 (40.0)	64 (54.2)
2	71 (28.6)	46 (35.4)	25 (21.2)
3	39 (15.7)	21 (16.2)	18 (15.3)
>3	22 (8.9)	11 (8.5)	11 (9.3)
**Gestation (weeks), median (IQR)**	40 (35-40)	40 (35-40)	40 (35-40)
**Gestation (weeks)**			
<30	14 (5.6)	8 (6.2)	6 (5.1)
30-40	228 (91.9)	119 (91.5)	109 (92.4)
>40	5 (2.0)	3 (2.3)	2 (1.7)
Not recorded	1 (0.4)	0 (0)	1 (0.8)
**Birth Weight, in grams, median (IQR)**	2870 (2445-3270)	2835 (2400-3320)	2902 (2500-3240)
**Birth Weight (g)**			
very low/low (500-2499)	56 (22.6)	31 (23.8)	25 (21.2)
normal (2500-3900)	142 (57.3)	66 (50.8)	76 (64.4)
>3900	4 (1.6)	2 (1.5)	2 (1.7)
unknown	46 (18.5)	31 (23.8)	15 (12.7)
**Time known to ANC services (days) (%)**			
0-30	83 (33.5)	33 (25.4)	50 (42.4)
31-120	70 (28.2)	41 (31.5)	29 (24.6)
≥ 121	95 (38.3)	56 (43.1)	39 (33.1)
**Pregnancy Outcome (%)**			
Good	141 (56.9)	66 (50.8)	75 (63.6)
Adverse	74 (29.8)	40 (30.8)	34 (28.8)
Unknown*	33 (13.3)	24 (18.5)	9 (7.6)

ANC: antenatal care, DR: drug-resistant; DS: drug-susceptible; DST: drug-susceptible test; PHC: primary health care

*Excluded from regression model used to predict adverse pregnancy outcomes, as there was missing data for one or more of the following variables; live birth status, birth weight or gestational age.

For peer review only

Table 3: Multivariable logistic regression model for predictors of adverse pregnancy outcomes among women with tuberculosis (n = 215).

(a), (b), and (c) not added simultaneously to final model to avoid collinearity

		Total	Adverse	%	OR (95%CI)	aOR (95%CI)
Age category	14-17 years	8	2	25.0%	0.76 (0.15-3.96)	0.5 (0.05-5.24)
	18-24 years	64	21	32.8%	1.12 (0.58-2.15)	1.56 (0.65-3.76)
	25-34 years	115	35	30.4%	reference	
	35-44 years	28	16	57.1%	3.05 (1.31-7.11)	3.99 (1.37-11.57)
HIV status	HIV+	106	40	37.7%	1.34 (0.76-2.35)	2.72 (1.2-6.18)
	HIV-	109	34	31.2%	reference	
Location of diagnosis	Hospital	64	32	50.0%	2.6 (1.42-4.76)	2.14 (0.99-4.63)
	PHC	151	42	27.8%	reference	
Pervious TB treatment history	Retreatment	55	20	36.4%	1.19 (0.62-2.29)	0.7 (0.31-1.57)
	New	142	46	32.4%	reference	
Diagnostic method (a)	Bacteriologically confirmed	168	64	38.1%	2.28 (1.06-4.89)	2.33 (0.86-6.29)
	Clinically diagnosed	47	10	21.3	reference	
Site of disease (a)	PTB	174	59	33.9%	reference	
	EPTB	37	14	37.8%	1.19 (0.57-2.47)	
DST (a)	DR TB	8	4	50.0%	1.7 (0.41-7.05)	
	DST not recorded	56	14	25.0%	0.57 (0.28-1.13)	
	DS TB	151	56	37.1%	reference	
TB treatment linkage (b)	Linked within 30 days	172	52	30.2%	reference	
	Linked after 30 days	19	6	31.6%	1.07 (0.38-2.96)	
	Never linked	24	16	66.7%	4.62 (1.86-11.45)	
TB treatment outcome (b)	Unfavourable	68	32	47.1%	2.22 (1.23-4.03)	2.29 (1.03-5.08)
	Favourable	147	42	28.6%	reference	
Time attending antenatal services	0-30 days	71	36	50.7%	4.87 (2.36-10.06)	10.57 (4.01-27.89)
	31-120 days	58	23	39.7%	3.11 (1.45-6.69)	3.54 (1.39-8.98)
	≥121 days	86	15	17.4%	reference	
Parity (c)	continuous				0.7 (0.52-0.95)	
Gravidity (c)	continuous				0.7 (0.53-0.94)	0.55 (0.37-0.81)

For peer review only - http://bmjopen.bmj.com/site/about/guidelines.xhtml

For peer review only

For peer review only - http://bmjopen.bmj.com/site/about/guidelines.xhtml

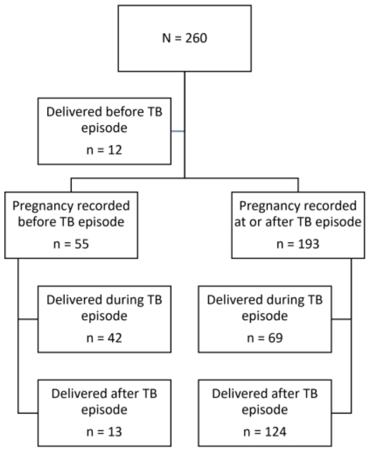

Figure 1: Timing of pregnancy in relation to the tuberculosis episode

863x484mm (130 x 130 DPI)

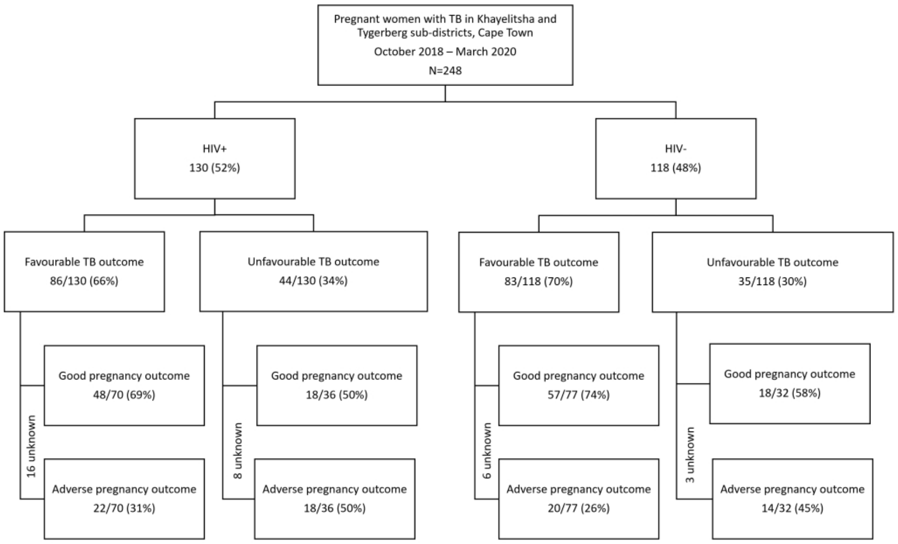

Figure 2: Tuberculosis outcomes and pregnancy outcomes by HIV status (n=248)

860x484mm (130 x 130 DPI)

The association between tuberculosis and pregnancy outcomes: A retrospective cohort study of women in Cape Town, South Africa.

Authors

Sue-Ann Meehan[1*], Anneke C. Hesseling[1], Arne von Delft[2,3], Florian M. Marx[1,4], Jennifer A. Hughes[1], Peter Bock[1], Aduragbemi Banke-Thomas[5,6], Rory Dunbar[1], Florence Phelanyane[2,3], Mariette Smith[2,3], Muhammad Osman[1,5]

*Corresponding author

Email address: sueannm@sun.ac.za (SM)

Affiliations:

[1]Desmond Tutu TB Centre, Department of Paediatrics and Child Health, Faculty of Medicine and Health Sciences, Stellenbosch University

[2]Centre for Infectious Disease Epidemiology and Research (CIDER), School of Public Health and Family Medicine, Faculty of Health Sciences, University of Cape Town

[3]Health Intelligence Directorate, Western Cape Government, Department of Health and Wellness

[4]Division of Infectious Disease and Tropical Medicine, Centre for Infectious Diseases, Heidelberg University Hospital, Heidelberg, Germany

[5]School of Human Sciences, Faculty of Education, Health and Human Sciences, University of Greenwich, London, United Kingdom

[6]Faculty of Epidemiology and Population Health, London School of Hygiene and Tropical Medicine, London, United Kingdom

For peer review only - http://bmjopen.bmj.com/site/about/guidelines.xhtml

For peer review only

Supplementary Table S1: Demographic and clinical characteristics among pregnant women with tuberculosis and HIV, stratified by pregnancy status at the time antiretroviral treatment was initiated (n=130).

	Total N (%)	Pregnant at ART start n (%)	
	**(N=130)**	**Yes (n=56), 43%**	**No (n=74), 57%**
**CD4 count*, median (IQR)**	235 (127-423)	231 (100-352)	260 (134-468)
**Age**			
14-17 years	3 (2.3)	0 (0)	3 (4.1)
18-24 years	25 (19.2)	9 (16.1)	16 (21.6)
25-34 years	82 (63.1)	39 (69.6)	43 (58.1)
35-44 years	20 (15.4)	8 (14.3)	12 (16.2)
**Pregnancy recorded in relation to TB diagnosis**			
Pregnant before TB diagnosed	33 (25.4)	14 (25.0)	19 (25.7)
Pregnant after TB diagnosed	97 (74.6)	42 (75.0)	55 (74.3)
**TB treatment at ART start**			
yes	37 (28.5)	21 (37.5)	16 (21.6)
no	93 (71.5)	35 (62.5)	58 (78.4)
**Viral suppression ever**			
yes	115 (88.5)	48 (85.7)	67 (90.5)
no	15 (11.5)	8 (14.3)	7 (9.5)
**Last VL suppressed**			
yes	97 (74.6)	42 (75.0)	55 (74.3)
no	33 (25.4)	14 (25.0)	19 (25.7)
**Time known to ANC services (days) (%)**			
0-30	33 (25.4)	17 (30.4)	16 (21.6)
31-120	41 (31.5)	19 (33.9)	22 (29.7)
≥ 121	56 (43.1)	20 (35.7)	36 (48.6)
**Pregnancy Outcome (%)**			
Good	66 (50.8)	30 (53.6)	36 (48.6)
Adverse	40 (30.8)	17 (30.4)	23 (31.1)
unknown	24 (18.5)	9 (16.1)	15 (20.3)

*CD4 count closest to date of TB diagnosis

ART: antiretroviral treatment, VL: viral load, ANC: antenatal care

For peer review only

For peer review only - http://bmjopen.bmj.com/site/about/guidelines.xhtml

STROBE Statement—Checklist of items that should be included in reports of ***cohort studies***

	Item No	Recommendation	Page No
**Title and abstract**	1	(*a*) Indicate the study's design with a commonly used term in the title or the abstract	1
		(*b*) Provide in the abstract an informative and balanced summary of what was done and what was found	2
**Introduction**			
Background/rationale	2	Explain the scientific background and rationale for the investigation being reported	4
Objectives	3	State specific objectives, including any prespecified hypotheses	4
**Methods**			
Study design	4	Present key elements of study design early in the paper	5
Setting	5	Describe the setting, locations, and relevant dates, including periods of recruitment, exposure, follow-up, and data collection	5
Participants	6	(*a*) Give the eligibility criteria, and the sources and methods of selection of participants. Describe methods of follow-up	5
		(*b*) For matched studies, give matching criteria and number of exposed and unexposed	N/A
Variables	7	Clearly define all outcomes, exposures, predictors, potential confounders, and effect modifiers. Give diagnostic criteria, if applicable	6 and Table 1
Data sources/ measurement	8*	For each variable of interest, give sources of data and details of methods of assessment (measurement). Describe comparability of assessment methods if there is more than one group	6
Bias	9	Describe any efforts to address potential sources of bias	6-7
Study size	10	Explain how the study size was arrived at	5
Quantitative variables	11	Explain how quantitative variables were handled in the analyses. If applicable, describe which groupings were chosen and why	6
Statistical methods	12	(*a*) Describe all statistical methods, including those used to control for confounding	8
		(*b*) Describe any methods used to examine subgroups and interactions	N/A
		(*c*) Explain how missing data were addressed	8
		(*d*) If applicable, explain how loss to follow-up was addressed	N/A
		(*e*) Describe any sensitivity analyses	N/A
**Results**			
Participants	13*	(a) Report numbers of individuals at each stage of study—eg numbers potentially eligible, examined for eligibility, confirmed eligible, included in the study, completing follow-up, and analysed	6-7
		(b) Give reasons for non-participation at each stage	N/A
		(c) Consider use of a flow diagram	26/27
Descriptive data	14*	(a) Give characteristics of study participants (eg demographic, clinical, social) and information on exposures and potential confounders	9-10
		(b) Indicate number of participants with missing data for each variable of interest	10
		(c) Summarise follow-up time (eg, average and total amount)	N/A
Outcome data	15*	Report numbers of outcome events or summary measures over time	9-10

For peer review only

Main results	16	(*a*) Give unadjusted estimates and, if applicable, confounder-adjusted estimates and their precision (eg, 95% confidence interval). Make clear which confounders were adjusted for and why they were included	10
		(*b*) Report category boundaries when continuous variables were categorized	8-10
		(*c*) If relevant, consider translating estimates of relative risk into absolute risk for a meaningful time period	N/A
Other analyses	17	Report other analyses done—eg analyses of subgroups and interactions, and sensitivity analyses	N/A
**Discussion**			
Key results	18	Summarise key results with reference to study objectives	11
Limitations	19	Discuss limitations of the study, taking into account sources of potential bias or imprecision. Discuss both direction and magnitude of any potential bias	15
Interpretation	20	Give a cautious overall interpretation of results considering objectives, limitations, multiplicity of analyses, results from similar studies, and other relevant evidence	11-14
Generalisability	21	Discuss the generalisability (external validity) of the study results	15
**Other information**			
Funding	22	Give the source of funding and the role of the funders for the present study and, if applicable, for the original study on which the present article is based	22

*Give information separately for exposed and unexposed groups.

Note: An Explanation and Elaboration article discusses each checklist item and gives methodological background and published examples of transparent reporting. The STROBE checklist is best used in conjunction with this article (freely available on the Web sites of PLoS Medicine at http://www.plosmedicine.org/, Annals of Internal Medicine at http://www.annals.org/, and Epidemiology at http://www.epidem.com/). Information on the STROBE Initiative is available at http://www.strobe-statement.org.

For peer review only

For peer review only - http://bmjopen.bmj.com/site/about/guidelines.xhtml

BMJ Open

The association between tuberculosis and pregnancy outcomes: A retrospective cohort study of women in Cape Town, South Africa.

Journal:	*BMJ Open*
Manuscript ID	bmjopen-2023-081209.R2
Article Type:	Original research
Date Submitted by the Author:	04-Jan-2024
Complete List of Authors:	Meehan, S; Stellenbosch University Faculty of Medicine and Health Sciences, Desmond Tutu TB Centre, Department of Paediatrics and Child Health<br>Hesseling, A C; Stellenbosch University Faculty of Medicine and Health Sciences, Desmond Tutu TB centre, Department of Paediatrics and Child Health<br>von Delft, Arne; University of Cape Town Department of Medicine, Centre for Infectious Disease Epidemiology and Research (CIDER), School of Public Health and Family Medicine; Western Cape Department of Health, Health Intelligence Directorate<br>Marx, Florian M.; Stellenbosch University Faculty of Medicine and Health Sciences, Desmond Tutu TB centre, Department of Paediatrics and Child Health; Heidelberg university hospital, Division of Infectious Disease and Tropical Medicine, Centre for Infectious Diseases<br>Hughes, Jennifer A.; Stellenbosch University, Desmond Tutu TB Centre, Department of Paediatrics and Child Health<br>Bock, Peter; Stellenbosch University Faculty of Medicine and Health Sciences, Desmond Tutu TB Centre, Department of Paediatrics and Child Health<br>Banke-Thomas, Aduragbemi; University of Greenwich; London School of Hygiene & Tropical Medicine, Faculty of Epidemiology and Population Health<br>Dunbar, R.; Stellenbosch University Faculty of Medicine and Health Sciences, Desmond Tutu TB Centre, Department of Paediatrics and Child Health<br>Phelanyane, Florence; University of Cape Town Faculty of Health Sciences, Centre for Infectious Disease Epidemiology and Research (CIDER), School of Public Health and Family Medicine; Western Cape Department of Health, Health Intelligence Directorate, Department of Health and Wellness<br>Smith, Mariette; Western Cape Department of Health, Health Intelligence Directorate, Department of Health and Wellness ; University of Cape Town Faculty of Health Sciences, Centre for Infectious Disease Epidemiology and Research (CIDER), School of Public Health and Family Medicine<br>Osman, Muhammad; Stellenbosch University Faculty of Medicine and Health Sciences, Desmond Tutu TB Centre, Department of Paediatrics and Child Health; University of Greenwich, School of Human Sciences, Faculty of Education, Health and Human Sciences

**Primary Subject Heading**:	Public health
Secondary Subject Heading:	Infectious diseases, Health services research
Keywords:	Tuberculosis < INFECTIOUS DISEASES, Maternal medicine < OBSTETRICS, HIV & AIDS < INFECTIOUS DISEASES

SCHOLARONE™ Manuscripts

I, the Submitting Author has the right to grant and does grant on behalf of all authors of the Work (as defined in the below author licence), an exclusive licence and/or a non-exclusive licence for contributions from authors who are: i) UK Crown employees; ii) where BMJ has agreed a CC-BY licence shall apply, and/or iii) in accordance with the terms applicable for US Federal Government officers or employees acting as part of their official duties; on a worldwide, perpetual, irrevocable, royalty-free basis to BMJ Publishing Group Ltd ("BMJ") its licensees and where the relevant Journal is co-owned by BMJ to the co-owners of the Journal, to publish the Work in this journal and any other BMJ products and to exploit all rights, as set out in our licence.

The Submitting Author accepts and understands that any supply made under these terms is made by BMJ to the Submitting Author unless you are acting as an employee on behalf of your employer or a postgraduate student of an affiliated institution which is paying any applicable article publishing charge ("APC") for Open Access articles. Where the Submitting Author wishes to make the Work available on an Open Access basis (and intends to pay the relevant APC), the terms of reuse of such Open Access shall be governed by a Creative Commons licence – details of these licences and which Creative Commons licence will apply to this Work are set out in our licence referred to above.

Other than as permitted in any relevant BMJ Author's Self Archiving Policies, I confirm this Work has not been accepted for publication elsewhere, is not being considered for publication elsewhere and does not duplicate material already published. I confirm all authors consent to publication of this Work and authorise the granting of this licence.

For peer review only

The association between tuberculosis and pregnancy outcomes: A retrospective cohort study of women in Cape Town, South Africa.

Authors

Sue-Ann Meehan[1*], Anneke C. Hesseling[1], Arne von Delft[2,3], Florian M. Marx[1,4], Jennifer A. Hughes[1], Peter Bock[1], Aduragbemi Banke-Thomas[5,6], Rory Dunbar[1], Florence Phelanyane[2,3], Mariette Smith[2,3], Muhammad Osman[1,5]

*Corresponding author

Email address: sueannm@sun.ac.za (SM)

Affiliations:

[1]Desmond Tutu TB Centre, Department of Paediatrics and Child Health, Faculty of Medicine and Health Sciences, Stellenbosch University

[2]Centre for Infectious Disease Epidemiology and Research (CIDER), School of Public Health and Family Medicine, Faculty of Health Sciences, University of Cape Town

[3]Health Intelligence Directorate, Western Cape Government, Department of Health and Wellness

[4]Division of Infectious Disease and Tropical Medicine, Centre for Infectious Diseases, Heidelberg University Hospital, Heidelberg, Germany

[5]School of Human Sciences, Faculty of Education, Health and Human Sciences, University of Greenwich, London, United Kingdom

[6]Faculty of Epidemiology and Population Health, London School of Hygiene and Tropical Medicine, London, United Kingdom

Running Head: Pregnancy outcomes among women with TB

Key words: Maternal health, pregnancy outcomes, Tuberculosis, outcomes, linkage to care, HIV

Journal: BMJ Open

Word count: Abstract: 299/300 Main text: 3999/4000

For peer review only - http://bmjopen.bmj.com/site/about/guidelines.xhtml

Abstract

Background

Tuberculosis (TB) remains a leading cause of mortality among women of childbearing age and a significant contributor to maternal mortality. Pregnant women with tuberculosis are at high risk of adverse pregnancy outcomes. This study aimed to determine risk factors for an adverse pregnancy outcome among pregnant women diagnosed with tuberculosis,

Methods

Using tuberculosis programmatic data, this retrospective cohort analysis included all women who were routinely diagnosed with tuberculosis in the public sector between October 2018 and March 2020 in two health sub-districts of Cape Town, and who were documented to be pregnant during their tuberculosis episode. Adverse pregnancy outcome was defined as either a live birth of an infant weighing <2500g and/or with a gestation period <37 weeks or as stillbirth, miscarriage, termination of pregnancy, maternal or early neonatal death. Demographics, TB and pregnancy characteristics were described by HIV status. Logistic regression was used to determine risk factors for adverse pregnancy outcome.

Results

Of 248 pregnant women, half (52%) were living with human immunodeficiency virus (HIV); all were on antiretroviral therapy (ART) at the time of their tuberculosis diagnosis. Pregnancy outcomes were documented in 215 (87%) women, of whom 74 (34%) had an adverse pregnancy outcome. Being older (35-44 years vs 25-34 years (aOR: 3.99; 95% CI:1.37-11.57), living with HIV (aOR: 2.72; 95%CI: 0.99-4.63), having an unfavourable TB outcome (aOR 2.29; 95% CI: 1.03-5.08), and having presented to antenatal services ≤1 month prior to delivery (aOR 10.57: 95% CI:4.01-27.89) were associated with higher odds of an adverse pregnancy outcome.

Conclusions

Pregnancy outcomes among women with TB were poor, irrespective of HIV status. Pregnant women with TB are a complex population who need additional support prior to, during and after TB treatment to improve TB treatment and pregnancy outcomes. Pregnancy status should be considered for inclusion in TB registries.

For peer review only

For peer review only - http://bmjopen.bmj.com/site/about/guidelines.xhtml

Strengths and Limitations

- Using the Provincial Health Data Centre (PHDC), enabled the use of routine programmatic data from multiple integrated sources, that allowed us to evaluate pregnancy and TB outcomes with consideration of HIV.
- The PHDC enabled the use of an expanded definition of ‘unfavourable TB treatment outcome’, which more accurately reflects ‘linkage to care’ within the TB program for pregnant women.
- We could not determine a pregnancy outcome for 13% of our cohort, due to missing data, which is a limitation when using routine data.
- The data extract used for this analysis included all persons with TB, which meant that we were not able to compare pregnancy outcomes for women who did not have TB.

For peer review only

For peer review only - http://bmjopen.bmj.com/site/about/guidelines.xhtml

Background

Globally, 10.6 million people developed tuberculosis (TB) in 2021, of whom 3.4 million (32.1%) were women(1). TB incidence peaks in women during the reproductive years,(2,3) is a leading cause of mortality among women of childbearing age(4) and a significant contributor to maternal mortality(5). Pregnant women with TB are at high risk of adverse pregnancy outcomes including prematurity, low birthweight and stillbirth(6–8).

TB is common in settings with high HIV prevalence, especially in sub-Saharan Africa. People living with human immunodeficiency virus (HIV) have a 2.5 times increased risk of developing TB despite access to antiretroviral therapy (ART),(9) and TB remains the leading cause of death among people living with HIV(10). Pregnant women with TB and HIV are at increased risk of adverse pregnancy outcomes(11,12) and higher maternal and infant mortality(13–16). There are limited data on TB treatment outcomes amongst pregnant women since pregnancy status is not routinely captured or reported as a TB programme indicator globally and pregnancy testing results are not routinely captured for women with TB. While ART(17) and TB preventive treatment (TPT)(18) reduces the risk of TB disease, an estimated 150,600 pregnant and 49,000 postpartum women develop TB annually, with women in Africa accounting for 40% of this burden(19). There is a need to integrate routine pregnancy indicators in TB programmes to improve surveillance of pregnant women with TB.

In high TB burden settings, the World Health Organization (WHO) recommends screening pregnant women for TB at every contact with a healthcare worker(20). In South Africa, routine testing of all pregnant women for TB and providing TPT once TB disease is excluded is recommended(18) but the extent of implementation is unknown and most likely incomplete. There is a pressing need for improved data from routine and programmatic sources to identify programmatic gaps and inform evidence-based research interventions to improve health outcomes for pregnant women with TB and their infants. This study used routine data across three health programs (TB, maternal health, and HIV services) to determine risk factors associated with an adverse pregnancy outcome for women with TB.

For peer review only - http://bmjopen.bmj.com/site/about/guidelines.xhtml

Methods

Setting

We used routine health data from two large health sub districts (Khayelitsha and Tygerberg) in the Cape Metro, Western Cape Province, South Africa. The Cape Metro has a population of approximately 4.6 million people (two thirds of the provincial population); HIV/AIDS and TB were among the top six causes of death in 2015(21). Antenatal HIV prevalence was 22% in 2019(22). There were 26,000 newly diagnosed persons with TB and more than 2,000 TB deaths in 2022(23). Maternal mortality ranged between 43.6 and 66.8 per 100 000 live births at healthcare facilities in 2017-2019(24). Khayelitsha is a peri-urban low resourced health sub district, with a mix of formal and informal housing (25). It is serviced by a district hospital and 10 primary healthcare (PHC) facilities(21). Tygerberg is a large health subdistrict, (25) with mostly formal dwellings. It has a tertiary hospital, a district level hospital and 21 PHC facilities(21).

Study design

This was a retrospective cohort study based on programmatic data collected as part of a larger health system strengthening study (LINKEDin), which aimed to reduce initial loss to follow-up for people diagnosed with TB in three South African provinces(26).

Study population

We included data for all women who were routinely diagnosed with TB at either a hospital or PHC facility in the two health subdistricts between October 2018 and March 2020 and who were documented as having a pregnancy that overlapped with their TB episode (See Table 1 for definition of key terms), as recorded in routine health records.

Data collection

Data were extracted from the Western Cape Provincial Health Data Centre (PHDC). The PHDC is a comprehensive linked health information exchange in the province and uses unique identifiers to integrate all electronic health data from routine health information systems in the public sector (laboratory, pharmacy, administrative and other clinical data) into single patient level records to support improved patient care(27). PHDC outputs include line lists (cascades) of patients with specific conditions e.g. TB, HIV, pregnancy(27). These are updated daily and can be used to track patients at each

For peer review only - http://bmjopen.bmj.com/site/about/guidelines.xhtml

healthcare visit and identify those requiring follow-up, including those newly diagnosed with TB who have not yet been entered into a TB treatment register and initiated on TB treatment.

We accessed the de-identified TB cascade from the PHDC and filtered for pregnancy status, to identify all women with TB who were pregnant during the study period in the two sub-districts. We used the unique identifier for these individuals to access data contained in the HIV and pregnancy cascades. Cascades are individual patient level views of specific health conditions which include key dates, evidence, outcomes, and co-morbidities relevant for patient management. We merged the three cascades and checked our dataset for consistency, confirming that all women included in our cohort had a pregnancy that overlapped with their TB episode (Table 1).

We extracted demographic (age) and clinical variables for TB, HIV, and pregnancy (e.g., *Mycobacterium tuberculosis* drug susceptibility status, HIV status, CD4 count, gravidity, infant birthweight etc) and key dates (date of TB diagnosis, date first known to the public antenatal services, date of delivery etc). To determine TB treatment outcomes, we reviewed the evidence recorded in the PHDC relating to evidence of linkage to a TB treatment facility and if linked, we extracted their TB treatment initiation and outcome data (cured, successfully completed, treatment failure, loss to follow up, died, not evaluated). We allocated a TB treatment outcome (favourable or unfavourable) as per our definition (Table 1). We used an expanded definition of unfavourable TB outcome to include newly diagnosed persons with TB who did not link to care and initiate treatment at a TB treatment facility. To determine a pregnancy outcome, we reviewed live birth, infant birthweight, and gestational period and allocated a pregnancy outcome (good or adverse), as per definitions in Table 1. Women who had one or more of these variables missing, had a pregnancy outcome of unknown asssigned. Pregnancy outcomes could occur either during or after the end of the TB episode (Figure 1).

Power calculation

As we were limited to a fixed sample size for this study, we implemented a pragmatic approach and considered the high proportion of adverse pregnancy outcomes in women with TB, ranging from 65% in a small cohort at a specialised hospital (15) to 27% in a

For peer review only - http://bmjopen.bmj.com/site/about/guidelines.xhtml

systematic review and meta-analysis of pregnant women with drug-resistant TB (6). With no data on pregnancy outcomes by TB treatment outcomes, we assumed a difference and estimated our sample of 250 pregnant women with TB would have 89% power with 95% significance to detect a difference of 20 percentage points in adverse pregnancy outcomes by TB outcomes.

Patient and Public involvement

As this study used routine data at the programme level, patients were not directly involved in any aspect of the study. We did engage our institutional community advisory board (CAB), on the design and implementation strategies of the broader overall study (LINKEDin), which primarily involved the evaluation of two interventions to reduce initial loss to follow up among TB patients in South Africa. In this sub analysis we used standardised TB and pregnancy outcomes. We presented our findings to the CAB prior to writing this manuscript and then again more recently to get their input on a proposed future study to address poor TB and pregnancy outcomes among this population.

Statistical analysis

Descriptive statistics were used to describe baseline characteristics, the timing of pregnancy and delivery compared to the TB episode, stratified by HIV status. A logistic regression model was used to determine risk factors for adverse pregnancy outcomes including TB treatment outcomes and other predictors. Predictors were added incrementally, observing the change in significance of the likelihood ratio test of each model, to produce a final adjusted model. The relationship between predictors was considered and we avoided co-linearity in the final model. SAS software (version 9.4; SAS Institute, Inc, Cary, NC) was used for analysis.

Ethical considerations

The Health Research Ethics Committee of Stellenbosch University (N18/07/069) approved the study, which was conducted according to the guiding principles within the Declaration of Helsinki. Approvals were received from the Western Cape Department of Health and the City of Cape Town Health Directorate. We received a waiver of individual consent for this routinely collected health data.

For peer review only - http://bmjopen.bmj.com/site/about/guidelines.xhtml

Results

Overall

A total of 260 pregnant women were diagnosed with TB between October 2018 and March 2020, of whom 67 had a pregnancy recorded prior to their TB diagnosis. We excluded 12 women because their pregnancy outcome was also recorded prior to their TB diagnosis. There were 111 women who had their pregnancy outcome documented before their TB outcome (i.e. who delivered while still on TB treatment), and 137 women who had their pregnancy outcome documented after their TB outcome (i.e. who delivered after their TB treatment). See Figure 1.

Among the 248 women included in analysis, the median age was 27.7 years (IQR: 22.7-32.3), range 14-43 years. The majority of women (191/248; 77%) had bacteriologically confirmed TB, 170/248 (69%) were treated for drug-susceptible TB and 10/248 (4%) were treated for drug-resistant TB. All had an HIV status recorded, half (130/248; 52%) were living with HIV. Women living with HIV (WLWH) were slightly older (29.1 years; IQR 25.7-33.4) compared to HIV negative women (25.4 years; IQR 20.6-30.1). Of WLWH, the median CD4 count at TB diagnosis was 236 cells/µL(IQR:127-423) and all were on antiretroviral therapy (ART) at the time of their TB diagnosis, with 56/130 (43%) having initiated ART during their pregnancy. Supplementary Table S1 provides characteristics of pregnant women with HIV by pregnancy status at the time ART was initiated. Overall, 9/248 of women (4%) died, of whom 6/9 (66%) were WLWH. Most women (6/9) died >60 days after their TB diagnosis had been made (Table 2).

For half of the women (121/248; 49%), this was their first pregnancy. Most (193/248; 78%) had their pregnancy recorded at or after the time their TB diagnosis was made, with 64% (124/193) delivering after their TB outcome was documented (Figure 1). A third (83/248; 33%) were documented to have attended antenatal services late, presenting for the first visit within 30 days of their delivery, with most of these women (67/83; 81%) presenting to services within two days of delivering their infant. Overall, the median gestation time was 40 weeks (IQR 35-40 weeks). Amongst infants overall, the median birth weight was 2875 grams (IQR:2445-3270 grams) (Table 2).

TB treatment outcomes

For peer review only - http://bmjopen.bmj.com/site/about/guidelines.xhtml

Using the more comprehensive definition including initial loss to follow-up, a third (79/248; 32%) of pregnant women had an unfavourable TB treatment outcome; 44/130 (33.8%) among WLWH and 35/118 (29.7%) among HIV negative women. Of those with an unfavourable TB treatment outcome, 34% (27/79) were never linked to TB care and were not recorded to have started TB treatment in a TB treatment register. Of those not linked to care, a quarter (7/27; 26%) died (3 HIV negative and 4 HIV positive) (Figure 2).

Pregnancy outcomes

Pregnancy outcomes were documented in 215/248 (86.7%) women. Overall, 74/215 (34.4%) women had an adverse pregnancy outcome; 40/106 (37.7%) among WLWH and 34/109 (31.2%) among HIV negative women. Overall, adverse pregnancy outcomes were higher among women who had unfavourable TB treatment outcomes (32/79; 40.5%) vs. favourable TB treatment outcomes (42/169; 24.9%), irrespective of HIV status (Figure 2).

Risk factors associated with an adverse pregnancy outcome

Pregnant women with TB were at increased odds of having an adverse pregnancy outcome if they were older [adjusted odds ratio (aOR) 3.99: 95% confidence interval (CI):1.37-11.57], living with HIV (aOR 2.72: 95%CI 1.2-6.18), had an unfavourable TB treatment outcome (aOR 2.29: 95%CI: 1.03-5.08) and if they presented late to antenatal services (≤30 days prior to their delivery), compared to attending antenatal services for at least 121 days (aOR 10.57: 95%CI:4.01-27.89). Women were less likely to have an adverse pregnancy outcome with increasing gravidity (aOR 0.55: 95%CI:0.37-0.81) (Table 3).

Discussion

This study included a large cohort of women diagnosed with TB in routine services with a pregnancy that overlapped their TB episode. Pregnancy outcomes were poor; being older, living with HIV, having an unfavourable TB treatment outcome and presenting late to antenatal services were all risk factors associated with an adverse pregnancy outcome. Half of these women were living with HIV, higher than the estimated antenatal HIV prevalence rate of 22% in the setting(22). All HIV-positive women were on ART; more than half (57%) were already on ART prior to their pregnancy, with the balance initiating ART during their pregnancy. Overall, these findings indicate good HIV screening and ART

For peer review only - http://bmjopen.bmj.com/site/about/guidelines.xhtml

initiation services generally and specifically among pregnant women attending public antenatal services. As comparison, approximately 54% of all people living with HIV were on ART in 2019 in Cape Town (28).

Overall, 34% of women with TB had an adverse pregnancy outcome. A systematic review of 69 studies (published 2009-2021), the majority of which were from low-and-middle-income countries, reported poor pregnancy outcomes for women with TB, with most studies reporting low birthweight and/or preterm labour and/or spontaneous abortions(29). A South African study reported 48% of women routinely treated for MDR/RR-TB during 2013 to 2017, had a poor pregnancy outcome(30), higher than our study, where the majority had drug-sensitive TB. Other South African studies with cohorts between 2011(15) and 2014(14) showed that of infants born to HIV-positive mothers with TB, between 20% and 70% had a gestation period <37 weeks and between 21% and 59% had a low birth weight. Since these previous studies, there have been many substantial advances and positive policy changes in our setting including universal HIV testing, universal ART rollout(31) and pregnant women being eligible for TB preventive therapy irrespective of HIV status(18). Yet our findings show similar high rates of poor pregnancy outcomes in women, irrespective of HIV status. This is highly concerning. Our data highlights the need for additional support and interventions for pregnant women with TB, including strategies to improve the integration of TB and maternal services to improve TB and pregnancy outcomes for these women. Additional work is needed to understand the care pathway for these women, barriers and enablers to care and determine gaps between policy and implementation. We also note that comparison across studies is challenging as the definition of an adverse pregnancy outcome varied by study. A standardized definition including all variables that affect both maternal and infant outcomes is needed.

Having a poor TB treatment outcome was associated with having a poor pregnancy outcome, which is biologically plausible. We are not aware of other studies that have reported on poor TB treatment outcomes as a risk factor for adverse pregnancy outcomes. A study among pregnant women with MDR/RR-TB found that 40% had an unfavourable TB treatment outcome, as per traditional WHO definitions(7). Our study found a third of women had an unfavourable TB outcome, but used a broader definition

For peer review only - http://bmjopen.bmj.com/site/about/guidelines.xhtml

to include those lost prior to registration/treatment initiation(32). We identified a high proportion of women who did not link to TB care; 43% among HIV-negative women and 27% among WLWH. For persons with TB in South Africa, approximately 12% among drug-sensitive(33) and 37% among drug-resistant(34) persons with TB are never started on treatment. Considering that the majority of our cohort had drug-sensitive TB, loss prior to registration/treatment initiation among our cohort was higher than what is reported for persons with TB overall. As these women are not recorded in TB treatment registers, their data is not included in routine recording and reporting, further excerbating the gaps in surveillance of TB in women of childbearing age.

Pregnant women diagnosed with TB are a complex population and require more intensive follow up to ensure better TB and pregnancy outcomes, and to reduce mortality. We recommend improved one-on-one communication by healthcare workers when referring these women to care(35). Skilled personnel should ensure that women understand their disease and the importance of linking to ongoing care and treatment. More broadly, health services should update contact details of persons with TB at each health visit, so that they can be easily traced if they do not link to TB care. This is especially important for pregnant women with TB, who may have additional barriers to accessing care. Routine antenatal and postnatal services should serve as an important trigger to link women to TB treatment following screening and testing. Being lost to the health system after a TB diagnosis may be the underlining reason for having an adverse pregnancy outcome. The association between TB and pregnancy outcomes should be further explored to better understand how these are associated.

Most women had their pregnancy recorded after they were diagnosed with TB. This is an interesting finding, as typically women are screened for TB during antenatal visits. This finding may be highlighting a need for improved sexual and reproductive health counselling with women who are accessing TB care in this setting. We are also mindful of the limitations of using routine data and that the capturing of the first date known to the antenatal services may not always be accurate. This finding should be interpreted with caution. Offering effective contraception to women at TB treatment initiation(29,36) and screening for pregnancy at each TB-related visit, with integration of reproductive health services as part of TB care is critical(37). Women should be made aware of their increased

For peer review only - http://bmjopen.bmj.com/site/about/guidelines.xhtml

risk of poorer pregnancy outcomes and need for additional care if they conceive during their TB episode. If adequate counselling and assessment of family planning at the start of TB treatment is being implemented as per local guidelines, and women continue to fall pregnant during their TB episode, then there could be other factors associated with this. Socio-behavioural research may elicit such factors and provide more person-centered understanding that could guide better implementation of exitsing or more patient-centered policies .

Living with HIV was found to be a risk factor for having an adverse pregnancy outcome, similar to previous studies. In our study, women had similar poor pregnancy outcomes irrespective of whether ART was started prior to or during pregnancy. A systematic review of 73 cohort studies, found that ART reduced the risk of adverse perinatal outcomes in pregnant women living with HIV, but the risk remained higher than in HIV-negative women(38). This review did not specify if cohorts had TB during their pregnancy. There exists limited data on HIV as a risk factor for adverse pregnancy outcomes among women with TB. A South African study from a tertiary hospital comparing TB-exposed infant outcomes by HIV-exposure, found a higher proportion of prematurity (70% vs 52%), low birth weight (59% vs 57%) and infant mortality (4 stillbirths and 6 neonatal deaths vs 0 deaths) among HIV-exposed infants compared to HIV-unexposed infants(15). In this study 64% of women were on ART, compared to our study where all women were on ART. ART may reduce the risk of adverse pregnancy outcomes, but for WLWH and who have TB overlapping their pregnancy, adverse pregnancy outcomes are high, irrespective of ART. {Pregnancy remains an excellent opportunity to start ART, the WHO recommends the immediate initiation of lifelong ART for all people living with HIV(39). Despite a universal test and treat policy for HIV, the findings from our study show that many women start ART only at antenatal presentation. An increase in community-based HIV testing and treatment to support earlier ART initiation is essential. ART initiation prior to pregnancy may reduce adverse pregnancy outcomes for these women and also TB treatment outcomes.

Our findings show that a third of women (25% of WLWH and 42% of HIV-negative women) were only documented to have sought care at public antenatal services during the month prior to delivery, highlighting the challenge of ‘late booking’. All antenatal, TB and HIV

For peer review only

For peer review only - http://bmjopen.bmj.com/site/about/guidelines.xhtml

services are free of direct charge in South Africa in the public sector. This proportion is higher than in the 2019 South African Antenatal survey, where only a small proportion of women (5.6%) attended their first antenatal visit in the last trimester of their pregnancy(40). In the Cape Town Metro, 75% of women attend antenatal services before 20 weeks(22). South Africa guidelines indicate that booking should be as early as possible (41) to ensure optimal antenatal care. Clearly, pregnant women with TB are an especially high-risk group overall. Those who book late should be tested for TB as a priority and offered TPT. Tracking pregnancy using ‘action lists’ may offer the opportunity for personnel in the TB programme to create awareness around the importance of adequate antenatal visits and urge these women to access maternal services earlier in their pregnancy.

For peer review only

We found that gravidity had a protective effect. This could be an example of the "healthy mother effect," where women who have had successful pregnancies in the past are more likely to have successful pregnancies in the future(42). This finding should be interpreted with caution.

Our study addresses a key yet neglected aspect of TB and maternal child health, using routine health data from multiple integrated sources that allowed us to evaluate pregnancy and TB treatment outcomes with consideration of HIV. We demonstrate poor pregnancy outcomes in pregnant women with TB, half of whom were also living with HIV. Using the PHDC strengthened our ability to determine more inclusive definitions of favourable and unfavourable TB treatment outcomes and enabled us to include all pregnant women diagnosed with TB, so we could determine both pre- and post-loss to follow up during TB care. Using our expanded definition of an ‘unfavourable’ TB treatment outcome, this study was able to reflect more accurately ‘linkage to care’ within the TB program for pregnant women. From a program perspective, having a maternal cascade is a benefit for the monitoring of the maternal and infant health program in services. Health services should allow for integrated tools to provide optimal care for patients across multiple programs: maternal, TB and HIV programs.

We could not determine a pregnancy outcome for 13% of our cohort, due to missing data. This may be due to women delivering outside the province, data linkage limitations

For peer review only - http://bmjopen.bmj.com/site/about/guidelines.xhtml

between mother and infant, or incomplete data capture, potentially resulting in a biased outcome and over-reporting of adverse pregnancy outcomes. Missing data is a limitation when using routine health data sources. A second limitation is that we were unable to compare pregnancy outcomes for our cohort to women who did not have TB during their pregnancy. This analysis was nested within a larger study and our data extract was restricted to persons with TB. Future work should compare pregnancy outcomes for women by TB status and TB outcomes by pregnancy status. A third limitation is that while the multivariable regression model was able to control for selected factors, residual confounding could have occurred due to other maternal factors (gestational diabetes, hypertension, anaemia) or socio-economic factors (poverty, substance use, educational status). These data were not available for analysis and should be included in future work. A further limitation is the inability to explain the time between presenting with TB and the timing of pregnancy.

Conclusions

Pregnancy outcomes among women with TB were poor, irrespective of HIV status. This is a major concern. TB care needs to be strenghtened for pregnant women and health services need to think through how best to optimise maternal services within the TB program. We used routine data to track women across the TB, HIV and maternal healthcare cascades, allowing for more inclusive definitions and analysis of linkage to care and TB treatment and pregnancy outcomes. Our study highlights the high proportion of women diagnosed with TB who never started TB treatment and the large proportion of women who only sought antenatal care services during the month prior to delivery. Pregnant women with TB are a complex population who need additional support prior to, during and after TB treatment to improve pregnancy outcomes, irrespective of HIV status. Going forward, recording of pregnancy status should be a key indicator piloted as part of TB treatment registries, as this would result in better quality data and offer opportunites for earlier intervention and support of pregnant and postpartum women with TB.

For peer review only - http://bmjopen.bmj.com/site/about/guidelines.xhtml

For peer review only

Figure legends

Figure 1: Timing of pregnancy in relation to the tuberculosis episode

Figure 2: Tuberculosis outcomes and pregnancy outcomes by HIV status (n=248)

Acknowledgements

We wish to acknowledge our implementing partners; the University of Cape Town and the Centre for Infectious Disease Epidemiology and Research (CIDER) in the Western Cape Province. We further acknowledge the staff at the Western Cape Provincial Health Data Centre (PHDC) for their invaluable assistance. We highly appreciate input from the health staff at the provincial, district and sub-district health offices and those at the facilities in which the study was implemented.

Contributorship statement

SM and MO planned and designed the study, were involved in data collection, extraction, and validation. MO produced the analysis. SM and MO interpreted the data.

SM wrote the first draft and finalised the submission based on author and peer reviewer input.

AvD, RD, FP and MS were involved in data collection, extraction, and validation, interpretation of results, reviewed the manuscript and provided critical input.

ACH, FM, JH, PB, AB-T were involved in data interpretation, reviewed the manuscript and provided critical input.

All authors have reviewed the final version of the manuscript and approve of its content and submission for publication.

Conflict of Interest

The authors have no conflict of interest to declare.

Funding

This research study and publication was supported by the Bill and Melinda Gates Foundation (BMGF), Investment INV- 007130. The contents are the responsibility of the authors and do not necessarily reflect the views of the BMGF. The funders had no role in study design, data collection and analysis, decision to publish, or preparation of the manuscript.

For peer review only - http://bmjopen.bmj.com/site/about/guidelines.xhtml

ACH is funded by the South African National Research Foundation SaRchi Chair in Paediatric Tuberculosis.

Data sharing statement

The de-identified dataset is available from the first author, on request. This analysis was nested within a broader study and the dataset is still being analysed within the broader study and will only be made public after the primary paper has been published.

Ethical statement

Stellenbosch University Health Reseach Ethics Committee (N18/07/069). The study included routine programmatic data and so did not include human participants directly.

For peer review only

For peer review only - http://bmjopen.bmj.com/site/about/guidelines.xhtml

References

1. World Heath Organization. Global tuberculosis report 2022 [Internet]. Geneva; Available from: https://www.who.int/teams/global-tuberculosis-programme/tb-reports/global-tuberculosis-report-2022
2. Deluca A, Chaisson R, Martinson N. Intensified case finding for tuberculosis in prevention of mother-to-child transmission programs: A simple and potentially vital addition for maternal and child health. J Acquir Immune Defic Syndr. 2009;50(2):196–9.
3. Gupta A, Nayak U, Ram M, Bhosale R, Patil S, Basavraj A, et al. Postpartum tuberculosis incidence and mortality among HIV-infected women and their infants in Pune, India, 2002-2005. Clin Infect Dis. 2007;45(2):241–9.
4. Zumla A, Bates M, Mwaba P. The neglected global burden of tuberculosis in pregnancy. Lancet Glob Heal [Internet]. 2014;2(12):e675–6. Available from: http://dx.doi.org/10.1016/S2214-109X(14)70338-9
5. Loto OM, Awowole I. Tuberculosis in pregnancy: A review. J Pregnancy. 2012;2012(379271).
6. Alene KA, Murray MB, van de Water BJ, Becerra MC, Atalell KA, Nicol MP, et al. Treatment Outcomes Among Pregnant Patients With Multidrug-Resistant Tuberculosis. JAMA Netw Open. 2022;5(6):e2216527.
7. Mokhele I, Jinga N, Berhanu R, Dlamini T, Long L, Evans D. Treatment and pregnancy outcomes of pregnant women exposed to second-line anti-tuberculosis drugs in South Africa. BMC Pregnancy Childbirth [Internet]. 2021;21(1):1–11. Available from: https://doi.org/10.1186/s12884-021-03956-6
8. Jana N, Barik S, Arora N, Singh AK. Tuberculosis in pregnancy: The challenges for South Asian countries. J Obstet Gynaecol Res. 2012;38(9):1125–36.
9. Yaghoubi A, Salehabadi S, Abdeahad H, Hasanian SM, Avan A, Yousefi M, et al. Tuberculosis, human immunodeficiency viruses and TB/HIV co-infection in pregnant women: A meta-analysis. Clin Epidemiol Glob Heal. 2020;8(4):1312–20.
10. Zumla A, Malon P, Henderson J, Grange JM. Impact of HIV infection on tuberculosis. Postgr Med J. 2000;76:259–68.
11. Rollins NC, Coovadia HM, Bland RM, Coutsoudis A, Bennish ML, Patel D, et al. Pregnancy outcomes in HIV-infected and uninfected women in rural and urban South Africa. J Acquir Immune Defic Syndr. 2007;44(3):321–8.

For peer review only - http://bmjopen.bmj.com/site/about/guidelines.xhtml

12. Arab K, Spence AR, Czuzoj-Shulman N, Abenhaim HA. Pregnancy outcomes in HIV-positive women: a retrospective cohort study. Arch Gynecol Obstet. 2017;295(3):599–606.

13. Calvert C, Ronsmans C. The contribution of HIV to pregnancy-related mortality: A systematic review and meta-analysis. Aids. 2013;27(10):1631–9.

14. Salazar-Austin N, Hoffmann J, Cohn S, Mashabela F, Waja Z, Lala S, et al. Poor Obstetric and Infant Outcomes in Human Immunodeficiency Virus-Infected Pregnant Women with Tuberculosis in South Africa: The Tshepiso Study. Clin Infect Dis. 2018;66(6):921–9.

15. Bekker A, Schaaf HS, Draper HR, Kriel M, Hesseling AC. Tuberculosis disease during pregnancy and treatment outcomes in HIV-infected and uninfected women at a referral Hospital in Cape Town. PLoS One. 2016;11(11):1–14.

16. Mnyani CN, McIntyre JA. Tuberculosis in pregnancy. BJOG An Int J Obstet Gynaecol. 2011;118(2):226–31.

17. Lawn SD, Harries AD, Williams BG, Chaisson RE, Losina E, Cock KM De, et al. Antiretroviral therapy and the control of HIV-associated tuberculosis. Will ART do it? Int J Tuberc Lung Dis. 2011;15(5):571–81.

18. South African National Department of Health. National Guidelines on the Treatment of Tuberculosis Infection [Internet]. 2023. Available from: https://www.knowledgehub.org.za/system/files/elibdownloads/2023-03/Health_Latent TB Infection_2023_web.pdf

19. Mafirakureva N. Revisiting the burden of TB in pregnant and post-partum women. In: International Journal of Tuberculosis and Lung Disease Abstract book 50th World Conference Hyderabad, India [Internet]. 2019. Available from: https://theunion.org/our-work/conferences/history-of-the-union-world-conference-on-lung-health/conference-abstract-books

20. World Health Oghanization. WHO consolidated guidelines on tuberculosis Module 2: Screening Systemic screening for tuberculosis disease [Internet]. World Health Organization. 2020. Available from: https://apps.who.int/iris/handle/10665/340255

21. Western Cape Government: Health. Cape Metro Health District Plan (2021/22 to 2023/24). 2021.

22. Woldesenbet, S.A., Lombard, C., Manda, S., Kufa, T., Ayalew, K., Cheyip M., and Puren A. The 2019 National Antenatal Sentinel HIV Survey, South Africa, National

For peer review only - http://bmjopen.bmj.com/site/about/guidelines.xhtml

Department of Health. 2021.

23. Western Cape Government Department of Health. Western Cape Provincial Tuberculosis (TB) Dashboard [Internet]. 2022 [cited 2022 Oct 15]. Available from: https://www.westerncape.gov.za/site-page/provincial-tb-dashboard

24. Kalk E, Heekes A, Slogrove AL, Phelanyane F, Davies MA, Myer L, et al. Cohort profile: the Western Cape Pregnancy Exposure Registry (WCPER). BMJ Open. 2022;12(6).

25. Western Cape Government Health Department. Circular H 102/2020 Covid-19 Population Data. Chief Dir Strateg Heal Support [Internet]. 2020;1–123. Available from: https://www.westerncape.gov.za/assets/departments/health/h_102_2020_covid-19_population_data.pdf

26. Meehan S-A, Hesseling AC, Boulle A, Chetty J, Connell L, Dlamini-Miti NJ, et al. Reducing initial loss to follow up among people with bacteriologically confirmed tuberculosis: LINKEDin, a quasi-experimental study in South Africa. Open Forum Infect Dis. 2023;December.

27. Boulle A, Heekes A, Tiffin N, Smith M, Mutemaringa T, Zinyakatira, N, Phelanyane F, et al. Data Centre Profile: The Provincial Health Data Centre of the Western Cape. Int J Popul Data Sci. 2019;0(October).

28. Western Cape Government Department of Health. Cape Metro District Health Plan 2018/19 -2020/21 [Internet]. 2018. Available from: http://www.health.gov.za/DHP/docs/DHP2018-21/Western_Cape/Metro_District_Health_Plan.pdf

29. Nguyen Y, McNabb KC, Farley JE, Warren N. Examining family planning and adverse pregnancy outcomes for women with active tuberculosis disease: a systematic review. BMJ Open. 2022;12(3):e054833.

30. Loveday M, Hughes J, Sunkari B, Master I, Hlangu S, Reddy T, et al. Maternal and Infant Outcomes among Pregnant Women Treated for Multidrug/Rifampicin-Resistant Tuberculosis in South Africa. Clin Infect Dis. 2021;72(7):1158–68.

31. South African National Department of Health. Circular: Implementation of the universal test and treat strategy for HIV positive patients and differentiated care for stable patients [Internet]. 2016. Available from: https://sahivsoc.org/Files/22 8 16 Circular UTT Decongestion CCMT Directorate.pdf

32. Linh NN, Viney K, Gegia M, Falzon D, Glaziou P, Floyd K, et al. World Health

For peer review only - http://bmjopen.bmj.com/site/about/guidelines.xhtml

Organization treatment outcome definitions for tuberculosis: 2021 update. Eur Respir J [Internet]. 2021;58(2). Available from: http://dx.doi.org/10.1183/13993003.00804-2021

33. Naidoo P, Theron G, Rangaka MX, Chihota VN, Vaughan L, Brey ZO, et al. The South African Tuberculosis Care Cascade: Estimated Losses and Methodological Challenges. J Infect Dis. 2017;216(Suppl 7):S702–13.

34. Cox H, Dickson-Hall L, Ndjeka N, van't Hoog A, Grant A, Cobelens F, et al. Delays and loss to follow-up before treatment of drug-resistant tuberculosis following implementation of Xpert MTB/RIF in South Africa: A retrospective cohort study. PLoS Med. 2017;14(2):1–19.

35. Viljoen L, Hendricks P, Hoddinott G, Vanqa N, Osman M, Hesseling AC, et al. Early interactions with newly diagnosed TB patients in hospital can support linkage to care. Public Heal Action [Internet]. 2022;12(3). Available from: http://dx.doi.org/10.5588/pha.22.0012%0A

36. Schnippel K, Ndjeka N, Conradie F, Berhanu R, Claasen Z, Banoo S, et al. A call to action: Addressing the reproductive health needs of women with drug-resistant tuberculosis. South African Med J. 2016;106(4):333–4.

37. Maugans C, Loveday M, Hlangu S, Waitt C, Schalkwyk M Van, Water B Van De, et al. Best practices for the care of pregnant people living with TB. Int J Lung Dis. 2023;27(January):357–66.

38. Portwood C, Sexton H, Kumarendran M, Brandon Z, Kirtley S, Hemelaar J. Adverse perinatal outcomes associated with antiretroviral therapy in women living with HIV: A systematic review and meta-analysis. Front Med. 2023;9(February).

39. World Heath Organization. Consolidated Guidelines on the use of Antiretroviral drugs for treating and preventing HIV infection. Recommendations for a public health approach. 2nd Edition. 2016.

40. Woldesenbet, S.A., Lombard, C., Manda, S., Kufa, T., Ayalew, K., Cheyip M., and Puren A. The 2019 National Antenatal Sentinel HIV Survey, South Africa, National Department of Health [Internet]. 2021. Available from: https://www.nicd.ac.za/wp-content/uploads/2021/11/Antenatal-survey-2019-report_FINAL_27April21.pdf

41. South African National Department of Health. Guidelines for maternity care in South Africa. A manual for clinics, community health centres and district hospitals. Fourth Edition. [Internet]. 2016. Available from:

For peer review only - http://bmjopen.bmj.com/site/about/guidelines.xhtml

https://knowledgehub.health.gov.za/elibrary/guidelines-maternity-care-south-africa-2016

42. Whitley E, Doyle P, Roman E, De Stavola B. The effect of reproductive history on future pregnancy outcomes. Hum Reprod. 1999;14(11):2863–7.

For peer review only

For peer review only - http://bmjopen.bmj.com/site/about/guidelines.xhtml

Table 1. Key variable definitions for TB, HIV, and pregnancy outcomes

**Definition of terminology**	
**TB episode**	Period from the date of TB diagnosis to the recorded date of TB outcome or death.
**TB diagnosis**	Includes a bacteriological (evidence of a positive TB laboratory test) or clinical diagnosis of TB (including evidence of TB drugs administered as part of a therapeutic regimen, or primary ICD10 codes and inclusion in a primary care TB register).
**Pregnancy episode**	The period from the first electronically captured evidence of a pregnancy documented in antenatal health services to the date of delivery or of any other pregnancy outcome.
**Overlapping pregnancy**	Date of the first evidence of the pregnancy was ≤9 months prior to the date of the TB diagnosis or ≤18 months after the date of the TB diagnosis
**TB treatment outcomes**	
Favourable TB outcome	Recorded in the routine health service data as treatment success (bacteriologically cured or successfully completed TB treatment).
Unfavourable TB outcome (expanded TB treatment outcome definition)	Recorded in the routine health service data as lost to follow up, died during treatment, treatment failed or not evaluated (WHO TB outcomes) OR loss to follow up prior to linkage to TB care (including died prior to linkage, i.e. "initial loss to follow-up").
**Linkage to TB care**	Evidence of accessing a TB treatment facility for TB treatment (TB hospital or a PHC facility offering TB treatment) after the diagnosis of TB.
**Pregnancy outcomes**	
Good pregnancy outcome	A pregnancy that resulted in the live birth of an infant weighing ≥2500g and with a gestation period ≥ 37 weeks.

For peer review only - http://bmjopen.bmj.com/site/about/guidelines.xhtml

Adverse pregnancy outcome	A pregnancy that resulted in either a live birth of an infant weighing <2500g and/or with a gestation period < 37 weeks or where there was a still birth, miscarriage, termination, the mother dying prior to delivery or early neonatal death.

For peer review only

For peer review only - http://bmjopen.bmj.com/site/about/guidelines.xhtml

Table 2. Demographic and clinical characteristics among pregnant women with tuberculosis, by HIV status (n=248).

	Total N (%)	HIV status n (%)	
	(N=248)	HIV+ (n=130)	HIV- (n=118)
**Age in years, median (IQR)**	27.7 (22.7-32.3)	29.1 (25.7-33.4)	25.4 (20.6-30.1)
14-17 years	10 (4.1)	3 (2.3)	7 (5.7)
18-24 years	75 (30.2)	25 (19.2)	50 (40.7)
25-34 years	129 (52.0)	82 (63.1)	47 (38.2)
35-44 years	34 (13.7)	20 (15.4)	14 (11.4)
**Pregnancy recorded in relation to TB diagnosis**			
Pregnant before TB diagnosed	55 (22.2)	33 (25.4)	22 (18.6)
Pregnant after TB diagnosed	193 (77.8)	97 (74.6)	96 (81.4)
**TB diagnosis (%)**			
Bacteriologically confirmed	191 (77.0)	93 (71.5)	98 (83.1)
Clinically diagnosed	57 (23.0)	37 (28.5)	20 (16.9)
**Place of TB diagnosis (%)**			
Hospital	78 (31.5)	46 (35.4)	32 (27.1)
PHC facility	170 (68.5)	84 (64.6)	86 (72.9)
**TB Treatment Category**			
New	162 (65.3)	85 (65.4)	77 (65.3)
Retreatment	66 (26.6)	37 (28.5)	29 (24.6)
Unknown	20 (8.1)	8 (6.2)	12 (10.2)
**Site of disease**			
Extra-pulmonary TB	44 (17.7)	32 (24.6)	12 (10.2)
Pulmonary TB	196 (79.0)	92 (70.8)	104 (88.1)
Not specified	8 (3.2)	6 (4.6)	2 (1.7)
**Drug susceptibility status**			
DR TB	10 (4.0)	5 (3.8)	5 (4.2)
DS TB	170 (68.5)	81 (62.3)	89 (75.4)
DST not recorded	68 (27.4)	44 (33.8)	24 (20.3)
**TB outcomes (%)**			
Favourable	169 (68.1)	86 (66.2)	83 (70.3)
Unfavourable	79 (31.9)	44 (33.8)	35 (29.7)
**Linked to TB Care**			
Linkage ≤30 days	202(81.5)	107 (82.3)	95 (80.5)
Linkage >30days	19 (7.7)	11 (8.5)	8 (6.8)
Unlinked	27 (10.9)	12 (9.2)	15 (12.7)
**Died**			
Yes	9 (3.6)	6 (4.6)	3 (2.5)
No	239 (96.4)	124 (95.4)	115 (97.5)
**Time to maternal death (from date of TB diagnosis)**			
0-30 days	3 (33.3)	1 (16.7)	2 (66.7)
30-59 days	0 (0)	0 (0)	0 (0)
61-90 days	2 (22.2)	2 (33.3)	0 (0)
≥90 days	4 (44.4)	3 (50.0)	1 (33.3)

For peer review only - http://bmjopen.bmj.com/site/about/guidelines.xhtml

For peer review only

**Parity**			
0	121 (48.8)	55 (42.3)	66 (55.9)
1	71 (28.6)	47 (36.2)	24 (20.3)
2	35 (14.1)	19 (14.63.8)	16 (13.6)
>2	21 (8.5)	9 (6.9)	12 (10.2)
**Gravidity**			
1	116 (46.8)	52 (40.0)	64 (54.2)
2	71 (28.6)	46 (35.4)	25 (21.2)
3	39 (15.7)	21 (16.2)	18 (15.3)
>3	22 (8.9)	11 (8.5)	11 (9.3)
**Gestation (weeks), median (IQR)**	40 (35-40)	40 (35-40)	40 (35-40)
**Gestation (weeks)**			
<30	14 (5.6)	8 (6.2)	6 (5.1)
30-40	228 (91.9)	119 (91.5)	109 (92.4)
>40	5 (2.0)	3 (2.3)	2 (1.7)
Not recorded	1 (0.4)	0 (0)	1 (0.8)
**Birth Weight, in grams, median (IQR)**	2870 (2445-3270)	2835 (2400-3320)	2902 (2500-3240)
**Birth Weight (g)**			
very low/low (500-2499)	56 (22.6)	31 (23.8)	25 (21.2)
normal (2500-3900)	142 (57.3)	66 (50.8)	76 (64.4)
>3900	4 (1.6)	2 (1.5)	2 (1.7)
unknown	46 (18.5)	31 (23.8)	15 (12.7)
**Time known to ANC services (days) (%)**			
0-30	83 (33.5)	33 (25.4)	50 (42.4)
31-120	70 (28.2)	41 (31.5)	29 (24.6)
≥ 121	95 (38.3)	56 (43.1)	39 (33.1)
**Pregnancy Outcome (%)**			
Good	141 (56.9)	66 (50.8)	75 (63.6)
Adverse	74 (29.8)	40 (30.8)	34 (28.8)
Unknown*	33 (13.3)	24 (18.5)	9 (7.6)

ANC: antenatal care, DR: drug-resistant; DS: drug-susceptible; DST: drug-susceptible test; PHC: primary health care

*Excluded from regression model used to predict adverse pregnancy outcomes, as there was missing data for one or more of the following variables; live birth status, birth weight or gestational age.

For peer review only - http://bmjopen.bmj.com/site/about/guidelines.xhtml

Table 3: Multivariable logistic regression model for predictors of adverse pregnancy outcomes among women with tuberculosis (n = 215).

(a), (b), and (c) not added simultaneously to final model to avoid collinearity

		Total	Adverse	%	OR (95%CI)	aOR (95%CI)
Age category	14-17 years	8	2	25.0%	0.76 (0.15-3.96)	0.5 (0.05-5.24)
	18-24 years	64	21	32.8%	1.12 (0.58-2.15)	1.56 (0.65-3.76)
	25-34 years	115	35	30.4%	reference	
	35-44 years	28	16	57.1%	3.05 (1.31-7.11)	3.99 (1.37-11.57)
HIV status	HIV+	106	40	37.7%	1.34 (0.76-2.35)	2.72 (1.2-6.18)
	HIV-	109	34	31.2%	reference	
Location of diagnosis	Hospital	64	32	50.0%	2.6 (1.42-4.76)	2.14 (0.99-4.63)
	PHC	151	42	27.8%	reference	
Pervious TB treatment history	Retreatment	55	20	36.4%	1.19 (0.62-2.29)	0.7 (0.31-1.57)
	New	142	46	32.4%	reference	
Diagnostic method (a)	Bacteriologically confirmed	168	64	38.1%	2.28 (1.06-4.89)	2.33 (0.86-6.29)
	Clinically diagnosed	47	10	21.3	reference	
Site of disease (a)	PTB	174	59	33.9%	reference	
	EPTB	37	14	37.8%	1.19 (0.57-2.47)	
DST (a)	DR TB	8	4	50.0%	1.7 (0.41-7.05)	
	DST not recorded	56	14	25.0%	0.57 (0.28-1.13)	
	DS TB	151	56	37.1%	reference	
TB treatment linkage (b)	Linked within 30 days	172	52	30.2%	reference	
	Linked after 30 days	19	6	31.6%	1.07 (0.38-2.96)	
	Never linked	24	16	66.7%	4.62 (1.86-11.45)	
TB treatment outcome (b)	Unfavourable	68	32	47.1%	2.22 (1.23-4.03)	2.29 (1.03-5.08)
	Favourable	147	42	28.6%	reference	
Time attending antenatal services	0-30 days	71	36	50.7%	4.87 (2.36-10.06)	10.57 (4.01-27.89)
	31-120 days	58	23	39.7%	3.11 (1.45-6.69)	3.54 (1.39-8.98)
	≥121 days	86	15	17.4%	reference	
Parity (c)	continuous				0.7 (0.52-0.95)	
Gravidity (c)	continuous				0.7 (0.53-0.94)	0.55 (0.37-0.81)

For peer review only - http://bmjopen.bmj.com/site/about/guidelines.xhtml

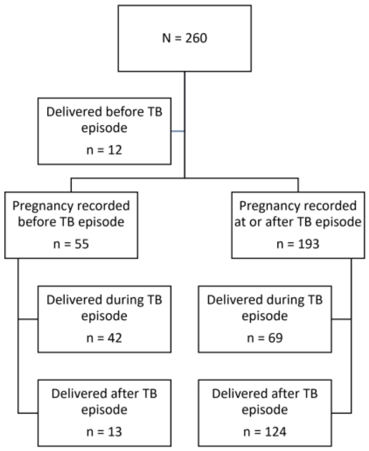

Figure 1: Timing of pregnancy in relation to the tuberculosis episode

863x484mm (130 x 130 DPI)

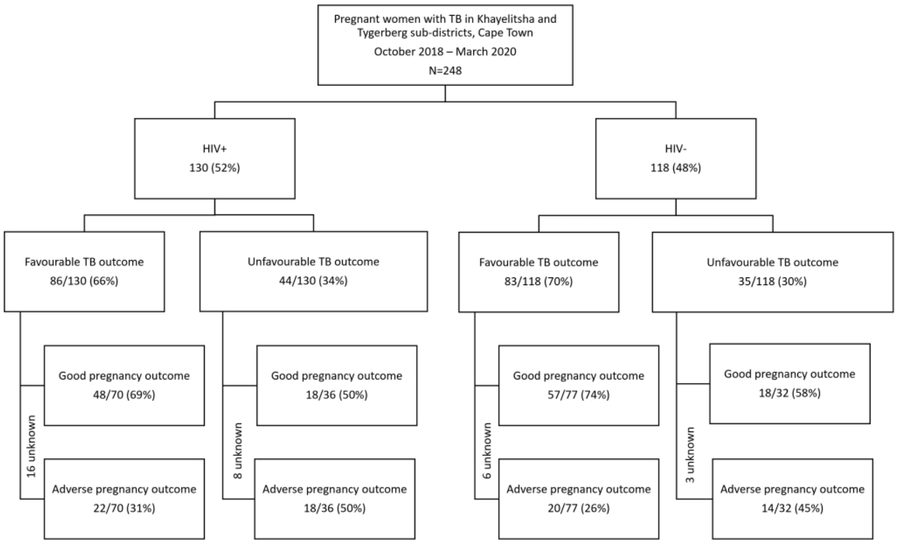

Figure 2: Tuberculosis outcomes and pregnancy outcomes by HIV status (n=248)

860x484mm (130 x 130 DPI)

The association between tuberculosis and pregnancy outcomes: A retrospective cohort study of women in Cape Town, South Africa.

Authors

Sue-Ann Meehan[1*], Anneke C. Hesseling[1], Arne von Delft[2,3], Florian M. Marx[1,4], Jennifer A. Hughes[1], Peter Bock[1], Aduragbemi Banke-Thomas[5,6], Rory Dunbar[1], Florence Phelanyane[2,3], Mariette Smith[2,3], Muhammad Osman[1,5]

*Corresponding author

Email address: sueannm@sun.ac.za (SM)

Affiliations:

[1]Desmond Tutu TB Centre, Department of Paediatrics and Child Health, Faculty of Medicine and Health Sciences, Stellenbosch University

[2]Centre for Infectious Disease Epidemiology and Research (CIDER), School of Public Health and Family Medicine, Faculty of Health Sciences, University of Cape Town

[3]Health Intelligence Directorate, Western Cape Government, Department of Health and Wellness

[4]Division of Infectious Disease and Tropical Medicine, Centre for Infectious Diseases, Heidelberg University Hospital, Heidelberg, Germany

[5]School of Human Sciences, Faculty of Education, Health and Human Sciences, University of Greenwich, London, United Kingdom

[6]Faculty of Epidemiology and Population Health, London School of Hygiene and Tropical Medicine, London, United Kingdom

For peer review only

Supplementary Table S1: Demographic and clinical characteristics among pregnant women with tuberculosis and HIV, stratified by pregnancy status at the time antiretroviral treatment was initiated (n=130).

	**Total N (%)**	**Pregnant at ART start n (%)**	
	**(N=130)**	**Yes (n=56), 43%**	**No (n=74), 57%**
**CD4 count*, median (IQR)**	235 (127-423)	231 (100-352)	260 (134-468)
**Age**			
14-17 years	3 (2.3)	0 (0)	3 (4.1)
18-24 years	25 (19.2)	9 (16.1)	16 (21.6)
25-34 years	82 (63.1)	39 (69.6)	43 (58.1)
35-44 years	20 (15.4)	8 (14.3)	12 (16.2)
**Pregnancy recorded in relation to TB diagnosis**			
Pregnant before TB diagnosed	33 (25.4)	14 (25.0)	19 (25.7)
Pregnant after TB diagnosed	97 (74.6)	42 (75.0)	55 (74.3)
**TB treatment at ART start**			
yes	37 (28.5)	21 (37.5)	16 (21.6)
no	93 (71.5)	35 (62.5)	58 (78.4)
**Viral suppression ever**			
yes	115 (88.5)	48 (85.7)	67 (90.5)
no	15 (11.5)	8 (14.3)	7 (9.5)
**Last VL suppressed**			
yes	97 (74.6)	42 (75.0)	55 (74.3)
no	33 (25.4)	14 (25.0)	19 (25.7)
**Time known to ANC services (days) (%)**			
0-30	33 (25.4)	17 (30.4)	16 (21.6)
31-120	41 (31.5)	19 (33.9)	22 (29.7)
≥ 121	56 (43.1)	20 (35.7)	36 (48.6)
**Pregnancy Outcome (%)**			
Good	66 (50.8)	30 (53.6)	36 (48.6)
Adverse	40 (30.8)	17 (30.4)	23 (31.1)
unknown	24 (18.5)	9 (16.1)	15 (20.3)

*CD4 count closest to date of TB diagnosis

ART: antiretroviral treatment, VL: viral load, ANC: antenatal care

For peer review only - http://bmjopen.bmj.com/site/about/guidelines.xhtml

STROBE Statement—Checklist of items that should be included in reports of ***cohort studies***

	Item No	Recommendation	Page No
**Title and abstract**	1	(*a*) Indicate the study's design with a commonly used term in the title or the abstract	1
		(*b*) Provide in the abstract an informative and balanced summary of what was done and what was found	2
**Introduction**			
Background/rationale	2	Explain the scientific background and rationale for the investigation being reported	4
Objectives	3	State specific objectives, including any prespecified hypotheses	4
**Methods**			
Study design	4	Present key elements of study design early in the paper	5
Setting	5	Describe the setting, locations, and relevant dates, including periods of recruitment, exposure, follow-up, and data collection	5
Participants	6	(*a*) Give the eligibility criteria, and the sources and methods of selection of participants. Describe methods of follow-up	5
		(*b*) For matched studies, give matching criteria and number of exposed and unexposed	N/A
Variables	7	Clearly define all outcomes, exposures, predictors, potential confounders, and effect modifiers. Give diagnostic criteria, if applicable	6 and Table 1
Data sources/ measurement	8*	For each variable of interest, give sources of data and details of methods of assessment (measurement). Describe comparability of assessment methods if there is more than one group	6
Bias	9	Describe any efforts to address potential sources of bias	6-7
Study size	10	Explain how the study size was arrived at	5
Quantitative variables	11	Explain how quantitative variables were handled in the analyses. If applicable, describe which groupings were chosen and why	6
Statistical methods	12	(*a*) Describe all statistical methods, including those used to control for confounding	8
		(*b*) Describe any methods used to examine subgroups and interactions	N/A
		(*c*) Explain how missing data were addressed	8
		(*d*) If applicable, explain how loss to follow-up was addressed	N/A
		(*e*) Describe any sensitivity analyses	N/A
**Results**			
Participants	13*	(a) Report numbers of individuals at each stage of study—eg numbers potentially eligible, examined for eligibility, confirmed eligible, included in the study, completing follow-up, and analysed	6-7
		(b) Give reasons for non-participation at each stage	N/A
		(c) Consider use of a flow diagram	26/27
Descriptive data	14*	(a) Give characteristics of study participants (eg demographic, clinical, social) and information on exposures and potential confounders	9-10
		(b) Indicate number of participants with missing data for each variable of interest	10
		(c) Summarise follow-up time (eg, average and total amount)	N/A
Outcome data	15*	Report numbers of outcome events or summary measures over time	9-10

For peer review only

Main results	16	(*a*) Give unadjusted estimates and, if applicable, confounder-adjusted estimates and their precision (eg, 95% confidence interval). Make clear which confounders were adjusted for and why they were included	10
		(*b*) Report category boundaries when continuous variables were categorized	8-10
		(*c*) If relevant, consider translating estimates of relative risk into absolute risk for a meaningful time period	N/A
Other analyses	17	Report other analyses done—eg analyses of subgroups and interactions, and sensitivity analyses	N/A
**Discussion**			
Key results	18	Summarise key results with reference to study objectives	11
Limitations	19	Discuss limitations of the study, taking into account sources of potential bias or imprecision. Discuss both direction and magnitude of any potential bias	15
Interpretation	20	Give a cautious overall interpretation of results considering objectives, limitations, multiplicity of analyses, results from similar studies, and other relevant evidence	11-14
Generalisability	21	Discuss the generalisability (external validity) of the study results	15
**Other information**			
Funding	22	Give the source of funding and the role of the funders for the present study and, if applicable, for the original study on which the present article is based	22

*Give information separately for exposed and unexposed groups.

Note: An Explanation and Elaboration article discusses each checklist item and gives methodological background and published examples of transparent reporting. The STROBE checklist is best used in conjunction with this article (freely available on the Web sites of PLoS Medicine at http://www.plosmedicine.org/, Annals of Internal Medicine at http://www.annals.org/, and Epidemiology at http://www.epidem.com/). Information on the STROBE Initiative is available at http://www.strobe-statement.org.

For peer review only
